# eQTL and multi-omics integration reveal PPIH as a prognostic and immunotherapeutic biomarker

**DOI:** 10.3389/fimmu.2025.1647722

**Published:** 2025-08-14

**Authors:** Fenglin Lv, Xinlu Zhang, Yanmei Wu, Zhipeng Li, Xiaomen Zheng, Huaxin Zhou, Wei Wang

**Affiliations:** ^1^ Department of Hepatobiliary Surgery, The Second Hospital of Shandong University, Jinan, China; ^2^ Shandong Province Engineering Research Center for Multidisciplinary Research on Hepatobiliary and Pancreatic Malignant Tumors, Jinan, China; ^3^ Department of Reproductive Medicine, Central Hospital Affiliated to Shandong First Medical University, Jinan, China; ^4^ Department of Obstetrics and Gynecology, Maternal and Child Health Care Hospital of Shandong Province, Jinan, China; ^5^ Medical Integration and Practice Center, Shandong University, Jinan, China

**Keywords:** PPIH, pan-cancer, multi-omics, tumor microenvironment, biomarker, hepatocellular carcinoma

## Abstract

**Background:**

Malignant tumors remain a major threat to global human health. This study aimed to systematically integrate multi-omics data to identify a candidate gene with biomarker potential across diverse cancer types and to evaluate its possible clinical applications in oncology.

**Methods:**

We first performed Mendelian randomization based on summary statistics to integrate blood expression quantitative trait loci data with genome-wide association study results from esophageal adenocarcinoma, stomach cancer, and clear cell renal cell carcinoma. A comprehensive series of multi-omics bioinformatics analyses was subsequently conducted to assess the gene’s expression patterns, genomic alterations, prognostic relevance, and associations with the tumor microenvironment (TME) across various cancer types. In addition, single-cell transcriptome data were analyzed to explore the gene’s functional roles in the TME. The key findings were further validated through *in vitro* experiments.

**Results:**

Mendelian randomization identified peptidylprolyl isomerase H (PPIH) as a potential biomarker across multiple malignancies. Single-cell transcriptome analysis suggested that this gene may enhance the proliferative ability of malignant cells and participate in communication between immune and stromal components in the TME. Multi-omics analyses revealed that the gene is abnormally expressed and significantly correlated with patient prognosis in several cancer types. Consistently, *in vitro* assays demonstrated that increased expression of PPIH promotes the proliferation, migration, and invasion of hepatocellular carcinoma (HCC) cells.

**Conclusion:**

This study highlights PPIH as a candidate biomarker with pan-cancer relevance and potential clinical value. These findings offer new directions for cancer diagnosis and provide a foundation for further development of targeted therapeutic approaches.

## 1 Introduction

Cancer has become the leading cause of premature death worldwide and is projected to surpass cardiovascular diseases as the primary cause of premature mortality in most countries during this century (1). According to the World Health Organization, approximately 10 million cancer-related deaths occurred globally in 2020 (1). Despite advancements in multimodal therapies, including surgery, chemotherapy, and targeted treatments, the five-year survival rate for patients with advanced-stage cancers remains dismal. For example, HCC has a post-treatment five-year recurrence rate as high as 70% (2), and distant metastasis occurs in 35–55% of cancer patients (3), both of which contribute significantly to poor clinical outcomes. Therefore, identifying novel biomarkers and therapeutic targets is critical to improving the overall prognosis for cancer patients.

The Summary data-based Mendelian Randomization (SMR) approach integrates summary statistics from genome-wide association studies (GWAS) with expression quantitative trait loci (eQTL) data to systematically prioritize candidate genes that may have a causal effect on disease phenotypes (4). Previous studies have demonstrated that SMR has notable advantages in distinguishing true causal genes from merely associated loci (5–9). However, most current SMR analyses have focused on individual cancer types, lacking a comprehensive investigation into common pathogenic genes across multiple cancers. Given that different cancer types may share similar genetic drivers during tumor initiation and progression (10, 11), pan-cancer multi-omics analyses based on SMR could facilitate the identification of key genes relevant to cancer development from a broader perspective.

In this study, we performed SMR analyses across multiple cancers originating from distinct organ systems to elucidate the causal relationships between specific genes and various malignancies. Specifically, we selected three histologically and anatomically distinct cancers—esophageal adenocarcinoma, stomach cancer, and clear cell renal cell carcinoma—and conducted SMR analyses to identify shared causal genes. Among the candidates, PPIH emerged as a potentially causal gene common to all three cancers. We then conducted a comprehensive pan-cancer characterization of PPIH using multi-omics data from public resources including The Cancer Genome Atlas (TCGA), Gene Expression Omnibus (GEO), XENA, and the Clinical Proteomic Tumor Analysis Consortium (CPTAC), aiming to assess its prognostic relevance and underlying molecular mechanisms. Cross-validation with analytical platforms such as GEPIA2, TIMER2, and UALCAN further reinforced the robustness and reliability of our findings. Additionally, we explored the pan-cancer landscape of PPIH through analyses of DNA methylation patterns, somatic mutation profiles, immune infiltration characteristics, protein–protein interaction networks, and functional enrichment pathways. Lastly, by integrating single-cell transcriptomic data, we investigated the regulatory role of PPIH within the TME, revealing its involvement in cancer cell proliferation and dynamic crosstalk between immune and stromal cells.

In conclusion, there is a lack of systematic research applying SMR approaches to investigate the causal roles of key genes in cancer susceptibility and progression from a pan-cancer perspective. This study represents a novel attempt to employ SMR for identifying cross-cancer oncogenes and characterizing their clinical and molecular features via multi-omics pan-cancer analyses. Our findings offer promising insights into the development of universal cancer biomarkers and therapeutic targets.

## 2 Methods

### 2.1 SMR analysis

To investigate the causal relationship between blood gene expression levels and a variety of cancers, GWAS and blood eQTL data analyzed by SMR were obtained from the GWAS Catalog, respectively (**Supplementary Table S1**). We included only gene expression probes containing at least one cis-eQTL with a P-value <5 × 10^−8^. The cis-eQTL with the lowest P-value was selected to distinguish it from other cis-eQTLs within the gene. For each probe, we tested the association between the trait and the probe using effect estimates from the eQTL study and the top cis-eQTL from the GWAS. The analysis process is implemented by SMR software version 1.03, with the default parameter recommended by the developer (27019110). For sensitivity analyses, we implemented the heterogeneity in dependent instruments (HEIDI) test to evaluate potential pleiotropic effects underlying observed gene-cancer associations, with significance thresholds being set at *pSMR < 0.05* and *pHEIDI > 0.05*. Specifically, SNPs in linkage disequilibrium (LD) with lead cis-eQTLs (r² > 0.9) were systematically excluded from HEIDI testing, as in nearly perfect LDS with top cis-EQTL, single nucleotide polymorphisms failed to provide information for the HEIDI test.

### 2.2 PPIH expression analysis

The Human Protein Atlas (HPA, https://www.proteinatlas.org/) provides comprehensive protein expression data in normal and cancerous tissues (12). We extracted PPIH expression profiles across diverse tissues from HPA. Tumor-specific expression patterns were analyzed using TIMER2 (http://timer.cistrome.org/) to compare PPIH levels between tumor and adjacent normal tissues across TCGA cohorts (13). To address the limited normal samples in TCGA, we integrated normal tissue data from the GTEx database. Violin plots were generated via the “Pathological Stage Plot” module in GEPIA2 illustrated PPIH expression dynamics across tumor stages (I–IV) using log2(TPM + 1)-transformed data. Differential expression of PPIH among molecular subtypes was further interrogated using TISIDB. Moreover, the protein expression profiles of PPIH across different cancer types were obtained from CPTAC database for protein-level validation.

### 2.3 Survival analysis

Univariable Cox regression analysis was performed using the “ezcox” R package to assess the prognostic impact of PPIH expression levels on four survival endpoints across all TCGA tumors: overall survival (OS), disease-specific survival (DSS), disease-free interval (DFI), and progression-free interval (PFI). Kaplan-Meier curves were generated via the “survminer” R package to compare median survival times between PPIH high and low expression cohorts, with statistical significance evaluated by the log-rank test.

### 2.4 Genetic alteration analysis

We systematically analyzed the genetic alteration landscape of PPIH via the cBioPortal (14) platform by selecting the “TCGA Pan-Cancer Atlas Study” via its “Quick Select” feature. The “Cancer Types Summary” provided detailed information of tumor-type-specific mutation frequencies, mutation spectra, and copy number alterations (CNAs) across TCGA cohorts. For immunological correlations, the TISIDB database (15) facilitated systematic exploration of associations between PPIH mutation profiles and both immune-related gene signatures and immune cell infiltrations.

### 2.5 Single-cell transcriptome analysis

All analyses in the present study were performed using R software (version 4.1.1). The scRNA-seq data were analyzed using Seurat package (version 4.2) (16). Cells with fewer than 500 genes and fewer than 1,000 total RNA counts were excluded from further analysis. We used harmony to correct for batch effects across datasets. Principal component analysis (PCA) were performed with the highly variable genes after Z-score normalization. t-Distributed Stochastic Neighbor Embedding (t-SNE) dimension reduction was performed with the top 20 significant principal components (PCs). Clusters were determined using the FindClusters function (resolution = 0.6). Malignant cells were identified using the CopyKAT algorithm, which estimates genome-wide copy number variations (CNVs) from scRNA-seq data. Cells with aneuploidy were classified as malignant, while diploid cells were considered normal. We analyzed intercellular communications using CellChat package (version 1.5.0) for scRNA-seq (17). The Monocle package was used to analyze the differentiation trajectory of hepatocytes into malignant cells and to generate a heatmap.

### 2.6 DNA methylation analysis

We obtained PPIH methylation profiles across TCGA tumors and systematically evaluated the correlation between site-specific promoter methylation levels and gene expression via Spearman’s correlation analysis. Heatmap visualization of methylation-expression relationships was generated via the “pheatmap” R package. Additionally, comparative analysis of pan-cancer promoter methylation differences between tumor and normal tissues was performed in UALCAN website.

### 2.7 Analysis of tumor microenvironment

We implemented systematic immune infiltration profiling through the “Immune-Gene” module of the TIMER2 website to interrogate the associations between PPIH expression and immune cell infiltrations across different TCGA malignancies. Immune cell fractions were quantified using a consortium of algorithms including TIMER, CIBERSORT, CIBERSORT-ABS, XCELL, and EPIC. Purity-adjusted Spearman’s rank correlation analysis was employed to compute partial correlation coefficients (cor) and the p values. The results were visualized in the form of heatmap.

### 2.8 Functional enrichment analysis

We first queried the STRING database (https://string-db.org/) to identify proteins interacting with PPIH. Subsequently, Gene Ontology (GO) enrichment analysis of the interacting genes was conducted using the “clusterProfiler” R package. In addition, the potential biological functions of PPIH were further explored through the Metascape platform. Finally, gene set enrichment analysis (GSEA) based on the Kyoto Encyclopedia of Genes and Genomes (KEGG) was performed in hepatocellular carcinoma (HCC) patients to identify biological pathways enriched in individuals with high or low PPIH expression.

### 2.9 Cell Culture

The human hepatocellular carcinoma (HCC) cell lines Huh7 and Hep3B were obtained from the Cell Bank of the Chinese Academy of Sciences (Shanghai, China). Cells were cultured under standardized conditions (37 °C, 5% CO_2_ humidified atmosphere) in DMEM medium (Gibco, Thermo Fisher Scientific) supplemented with 10% fetal bovine serum (FBS; Procell, China) and 1% penicillin-streptomycin solution. All cell lines were rigorously authenticated using short tandem repeat (STR) profiling and screened for mycoplasma contamination to ensure genetic fidelity and sterility.

### 2.10 Acquisition of clinical specimens

Primary hepatocellular carcinoma (HCC) tissues and matched adjacent non-tumorous liver tissues were obtained from patients who underwent surgical resection at the Second Hospital of Shandong University. All patients provided written informed consent prior to sample collection. The diagnosis of HCC was independently confirmed by two senior pathologists based on histopathological examination. This study was conducted in accordance with the Declaration of Helsinki and approved by the Ethics Committee of the Second Hospital of Shandong University. The collected tissue samples were used for Western blot analysis, qRT-PCR, and immunohistochemical staining, and all specimens were stored at −80°C in an ultra-low temperature freezer for subsequent experiments.

### 2.11 Lentiviral transduction and establishment of stable cell lines

Lentiviral vectors for PPIH overexpression and CRISPR/Cas9-mediated knockout were purchased from GeneChem (Shanghai, China). The overexpression lentivirus carried the full-length human PPIH coding sequence, while the knockout lentivirus was based on the CRISPR/Cas9 system targeting specific loci of the PPIH gene. Huh7 and Hep3B cells were seeded in 6-well plates and infected with the corresponding lentiviral particles when cell confluency reached 30%–50%. Polybrene (8 μg/mL; Santa Cruz Biotechnology, USA) was added to enhance transduction efficiency. After 48 hours of incubation, cells were subjected to puromycin (7 μg/mL; Solarbio, Beijing, China) selection for 5–7 days until all uninfected control cells were eliminated. To ensure experimental rigor and eliminate non-specific effects, appropriate negative controls were included. For overexpression experiments, an empty vector lentivirus (LV-NC) was used as a control. For CRISPR/Cas9 knockout, a non-targeting sgRNA control (sg-NC) was employed to account for any potential off-target or vector-related effects. The puromycin concentration and selection duration were optimized in advance through kill curve assays for both Huh7 and Hep3B cells, ensuring effective elimination of non-transduced cells without compromising the viability of stably transduced populations. Stably transduced cell lines with either PPIH overexpression or knockout were established and maintained in puromycin-containing medium. The efficiency of PPIH modulation was verified by quantitative reverse transcription PCR (qRT-PCR) and Western blot analysis.

### 2.12 RNA Extraction and qRT-PCR

Total RNA was extracted from cells using TRIzol reagent (Vazyme, Nanjing, China) according to the manufacturer’s instructions. RNA purity was assessed by measuring the 260/280 nm absorbance ratio using a spectrophotometer, and RNA integrity was confirmed by agarose gel electrophoresis. Genomic DNA was removed, and first-strand cDNA was synthesized using the HiScript II Q RT SuperMix (Vazyme). Quantitative real-time PCR (qRT-PCR) was performed using ChamQ Universal SYBR qPCR Master Mix (Vazyme) on an ABI QuantStudio system or equivalent platform. All reactions were run in triplicate, and β-actin was used as the internal control. Relative expression levels were calculated using the 2^−ΔΔCt method. The primer sequences used for qRT-PCR were as follows:

PPIH

Forward: 5′- CTGTGGTGATCTCGCAGTGT-3′

Reverse: 5′- CTTGATCAAATGGGGCAGCAG-3′

β-actin

Forward: 5′- CATGTACGTTGCTATCCAGGC-3′

Reverse: 5′- CTCCTTAATGTCACGCACGAT-3′

### 2.13 Western Blot Analysis

Cells were lysed using RIPA buffer (Beyotime, China) containing a protease inhibitor cocktail, and the supernatant was collected after centrifugation. Protein concentrations were measured using the BCA protein assay kit (Beyotime). Equal amounts of protein (20–30 μg per lane) were separated by SDS–polyacrylamide gel electrophoresis (SDS–PAGE) and transferred onto polyvinylidene difluoride (PVDF) membranes (Millipore, Bedford, MA, USA). Membranes were blocked with 5% non-fat milk at room temperature for 1 hour and incubated overnight at 4 °C with primary antibodies against PPIH and β-actin. After washing with TBST, membranes were incubated with horseradish peroxidase (HRP)-conjugated secondary antibodies for 2 hours at room temperature. Protein bands were visualized using an enhanced chemiluminescence (ECL) detection system (Vazyme, Nanjing, China). β-actin was used as the loading control. All antibodies used in the present study is listed in **Supplementary Table S2**.

### 2.14 Immunohistochemistry

Paraffin-embedded tissue sections (4 μm thickness) were mounted on glass slides, deparaffinized in xylene, and rehydrated through graded ethanol. Antigen retrieval was performed using citrate buffer, followed by blocking of endogenous peroxidase activity with 3% hydrogen peroxide. Sections were incubated with primary antibodies and subsequently with HRP-conjugated secondary antibodies using the UltraSensitive™ S-P kit (Beyotime, Shanghai, China) according to the manufacturer’s protocol. DAB was used for color development, and hematoxylin was used for counterstaining. The sections were dehydrated, mounted, and imaged.

### 2.15 Cell proliferation assay

Cell proliferation was measured using the Cell Counting Kit-8 (CCK-8; Vazyme, Nanjing, China). Cells were seeded into 96-well plates at a density of 2,000 cells per well and allowed to adhere. At the indicated time points, 10 μL of CCK-8 reagent was added to each well, followed by incubation for 2 hours in the dark. Absorbance at 450 nm was measured using a microplate reader to assess cell viability.

### 2.16 Colony formation assay

Log-phase cells were trypsinized, resuspended into single-cell suspensions, and seeded into 6-well plates at a density of 500 cells per well. After incubation for 10–14 days, with medium replaced as needed, cell colonies were fixed with 4% paraformaldehyde (Solarbio, Beijing, China) for 15 minutes and stained with 0.1% crystal violet for 30 minutes. Plates were gently washed, photographed, and the number of colonies was counted.

### 2.17 Transwell migration assay

Cell migration ability was assessed using Transwell chambers with 8 μm pore polycarbonate membranes (Corning, USA). Cells were resuspended in serum-free medium and seeded into the upper chambers at a density of 1 × 10^5^ cells per well. The lower chambers were filled with complete medium containing 10% fetal bovine serum (FBS; Procell, Wuhan, China) as a chemoattractant. After incubation at 37 °C with 5% CO_2_ for 48 hours, non-migrated cells on the upper surface were removed using cotton swabs. The migrated cells on the underside of the membrane were fixed with 4% paraformaldehyde (Solarbio) for 15 minutes and stained with 0.1% crystal violet. After washing, five random fields were imaged and the number of migrated cells was quantified.

### 2.18 EdU-555 cell proliferation assay

Log-phase cells were seeded into confocal culture dishes at a density of approximately 1.5 × 10^5^ cells per well and allowed to adhere for 12–16 hours. When cells reached ~80% confluency, they were incubated with EdU working solution from the BeyoClick™ EdU-555 Cell Proliferation Kit (C0075S, Beyotime, China) for 2 hours. After labeling, cells were fixed with 4% paraformaldehyde and washed with PBS containing 3% BSA. Permeabilization was performed using immunostaining permeabilization buffer (Beyotime) for 10–15 minutes. Cells were then incubated with the Click reaction mixture for 30 minutes at room temperature in the dark. Finally, nuclei were counterstained with DAPI (Solarbio), and images were acquired using a confocal microscope.

### 2.19 Wound Healing Assay

For the wound healing assay, 1 × 10^6^ cells per well were seeded into 6-well plates and allowed to adhere and grow until the cell monolayer reached approximately 90% confluency, which typically required 24 hours. The culture medium was then replaced with serum-free medium and cells were incubated for an additional 24 hours to suppress proliferation. A sterile 200 μL pipette tip was used to create a straight-line scratch through the cell monolayer. Detached cells were gently removed by washing twice with PBS. Images of the wound area were captured at 0, 24, 36, and 48 hours post-scratch using a phase-contrast microscope to evaluate cell migration and wound closure.

### 2.20 Statistical analysis

Statistical analyses were performed using GraphPad Prism 10 (GraphPad Software, San Diego, CA, USA). All results were based on at least three independent experiments and are presented as mean ± standard error of the mean (SEM). Chi-square test, Student’s t-test, and one-way analysis of variance (ANOVA) were used for parametric analyses. P-values are indicated as *p < 0.05, **p < 0.01, and ***p < 0.001. *A* P-value less than 0.05 was considered statistically significant.

## 3 Results

### 3.1 SMR analysis of blood eQTLs reveals pan-cancer candidate genes

**Figure 1:**
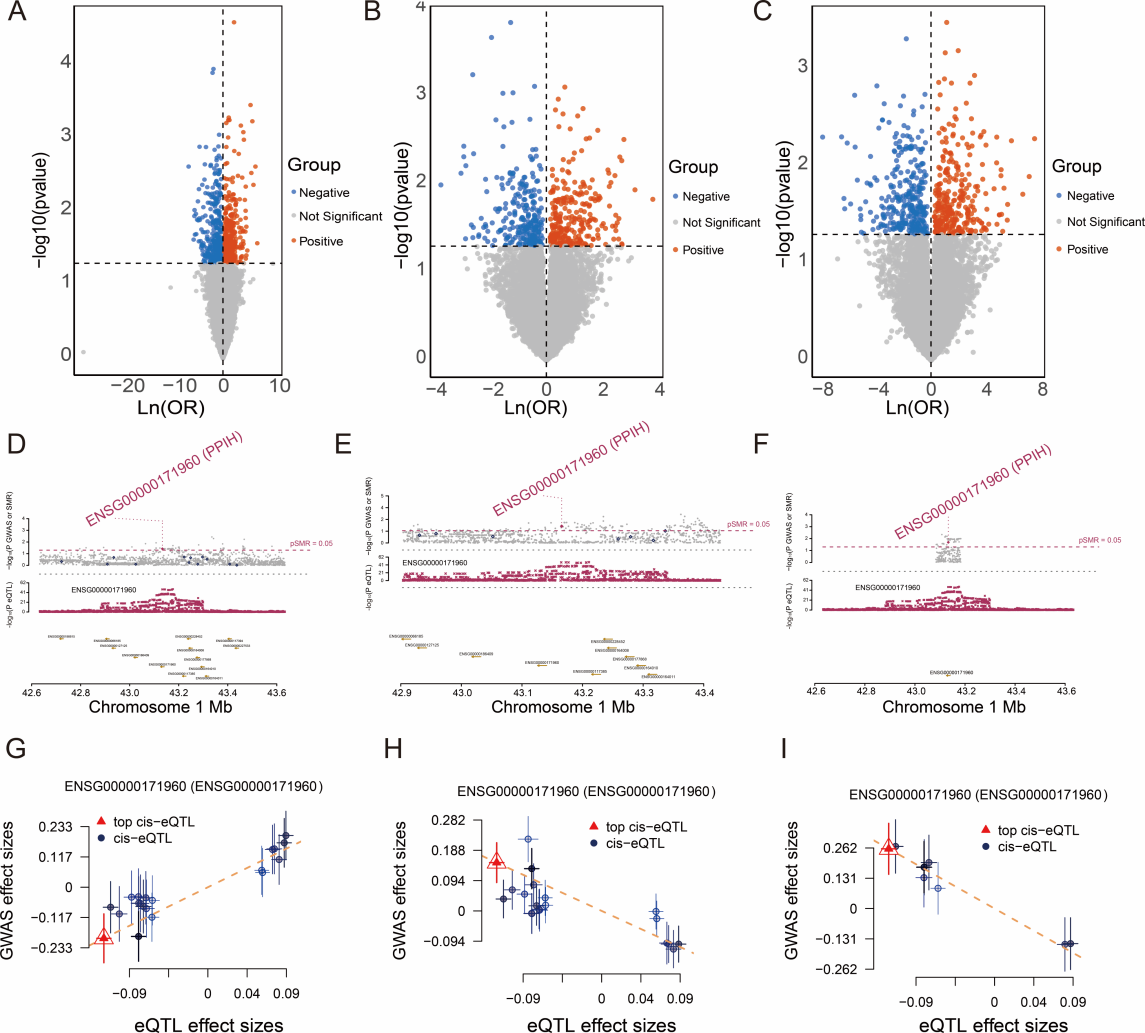
SMR Analysis of Cancers and the SMR effects of PPIH expression in blood on cancers. **(A–C)** The volcano plots illustrate the SMR results for esophageal carcinoma, kidney renal clear cell carcinoma, and stomach adenocarcinoma. **(D–F)** SMR locus plots show the association between PPIH and esophageal carcinoma, kidney renal clear cell carcinoma, and stomach adenocarcinoma. **(G–I)** SMR effect plots demonstrate the correlation between blood-derived PPIH expression and the risk of esophageal carcinoma, kidney renal clear cell carcinoma, and stomach adenocarcinoma.

In this study, we conducted a systematic analysis of established blood eQTL maps, which encompass cis-eQTL associations with gene expression derived from populations of American and European ancestry. To further investigate the potential pathogenic roles of these genes in malignancies, we performed SMR analysis on GWAS datasets of three distinct cancer types: esophageal cancer, gastric cancer, and clear cell renal cell carcinoma. Candidate genes were selected based on the criteria of P_SMR < 0.05 and P_HEIDI > 0.05. As shown in **Figures 1A–C**, a total of 674, 463, and 544 genes were identified to be significantly associated with esophageal cancer, clear cell renal cell carcinoma, and stomach cancer respectively. Notably, PPIH exhibited consistently significant associations across all three cancer types, suggesting that its expression levels may be closely linked to the risk of multiple cancers, thereby implying a potential role in tumorigenesis. To visually illustrate the SMR analysis results of PPIH, locus plots were generated for the three cancers (**Figures 1D–F**). Further characterization revealed that the cis-eQTL effects of PPIH showed a positive correlation with the GWAS effects in esophageal cancer but negative correlations in gastric cancer and clear cell renal cell carcinoma (**Figures 1G–I**). This divergent pattern suggests that PPIH may exert distinct mechanisms of action across different cancer types. The repeated significant association of PPIH across multiple cancer types suggests that it may serve as a key gene with pan-cancer relevance. In summary, our findings reveal a potential oncogenic role of PPIH in multiple malignancies and lay a foundation for further investigation of PPIH as a pan-cancer key gene.

### 3.2 Pan-cancer expression profile and clinical significance of PPIH

**Figure 2:**
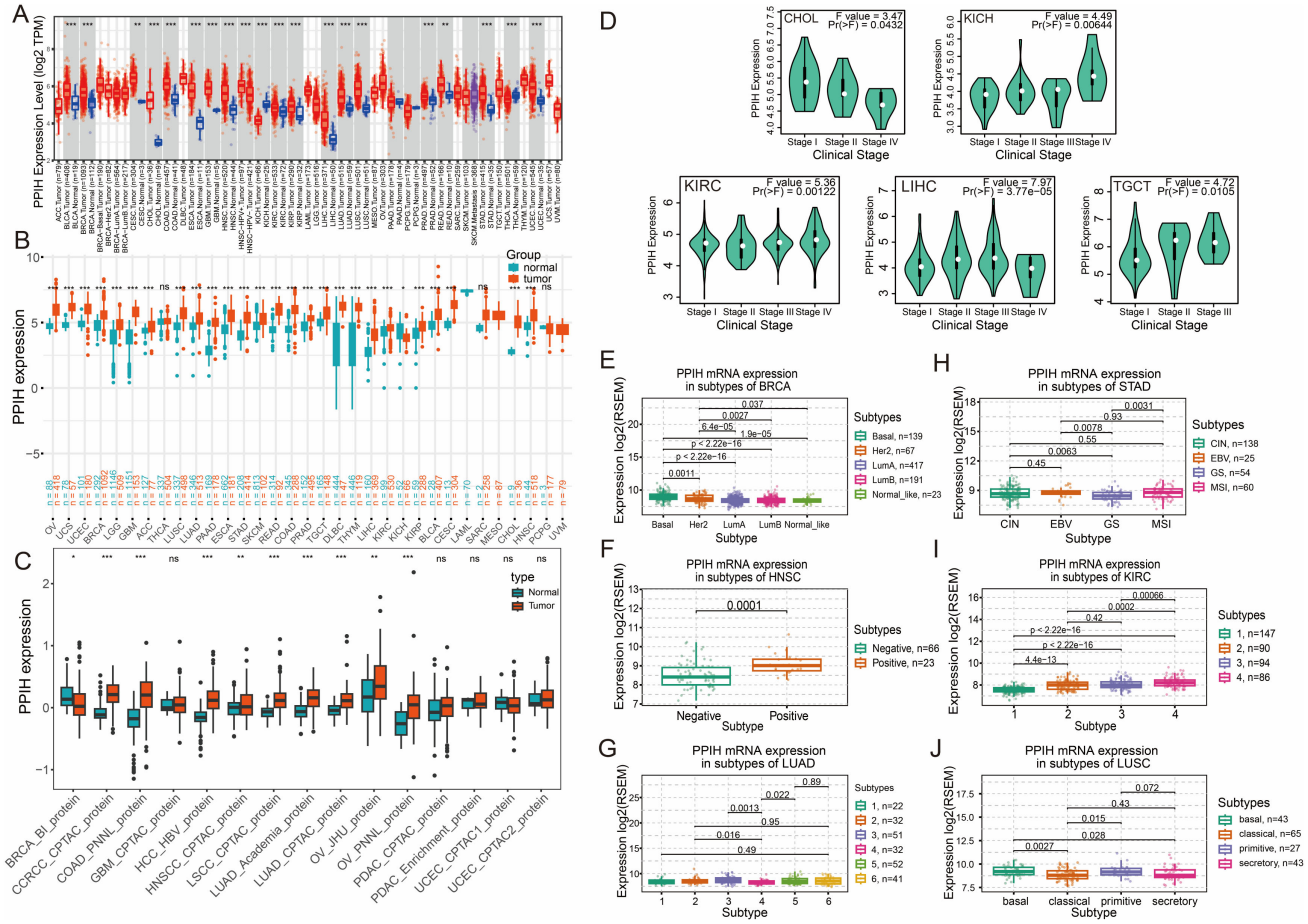
Pan-cancer expression analysis of PPIH. **(A)** Boxplots showing the aberrant RNA expressions of PPIH between tumor and normal tissues based on TIMER2 database. **(B)** Boxplots showing the aberrant RNA expressions of PPIH between tumor and normal tissues based on TCGA and GTEx database. **(C)** Boxplots showing the aberrant protein expressions of PPIH between tumor and normal tissues based on CPTAC database. **(D)** Violin plots showing the expression variations of PPIH across different tumor stages. **(E–J)** Violin plots showing the expression variations of PPIH across different tumor molecular subtypes. ns, not significant; *p < 0.05; **p < 0.01 and ***p < 0.001.

We performed a comprehensive pan-cancer analysis of PPIH expression using the TIMER2.0 platform. The results revealed that PPIH was significantly upregulated in various cancer types, including cholangiocarcinoma (CHOL), colorectal adenocarcinoma (COAD), esophageal carcinoma (ESCA), hepatocellular carcinoma (LIHC), and stomach adenocarcinoma (STAD) (**Figure 2A**). In addition, elevated PPIH expression was also observed in several tumors of other organ systems. Considering the limited number of normal tissue samples in the TCGA database, we incorporated the GTEx dataset to improve the reliability of the analysis. The integrated TCGA and GTEx data further confirmed that PPIH expression was consistently elevated in tumors compared with corresponding normal tissues (**Figure 2B**). Moreover, proteomic data from the CPTAC database demonstrated significant differences in PPIH protein levels between tumor and normal tissues across multiple cancer types (**Figure 2C**). Clinicopathological correlation analysis using the GEPIA2 platform revealed that PPIH expression was significantly associated with tumor stage in several cancer types, including CHOL, kidney chromophobe (KICH), kidney renal clear cell carcinoma (KIRC), LIHC, and testicular germ cell tumors (TGCT) (**Figure 2D**). Detailed tumor stage associations are provided in **Supplementary Figure S1**. Furthermore, molecular subtype analysis based on the TISIDB database uncovered subtype-specific expression patterns of PPIH. For instance, the basal-like subtype of breast cancer exhibited the highest PPIH expression; in head and neck squamous cell carcinoma, expression was significantly higher in HPV-positive cases than in HPV-negative ones; and in gastric cancer, the genomically stable (GS) subtype showed the lowest PPIH expression (**Figures 2E–J**). The elevated expression of PPIH across multiple cancer types suggests its potential involvement in key oncogenic processes. Its positive correlation with tumor stage in specific malignancies indicates that aberrant PPIH expression may be closely associated with tumor progression and aggressiveness. Moreover, the subtype-specific expression patterns observed across various cancers imply that PPIH may exert tissue- or context-dependent biological functions, providing a potential basis for patient stratification and precision therapy. Collectively, these findings underscore the clinical relevance of PPIH as both a biomarker and a putative oncogenic contributor in cancer.

### 3.3 Pan-cancer survival analysis of PPIH

**Figure 3:**
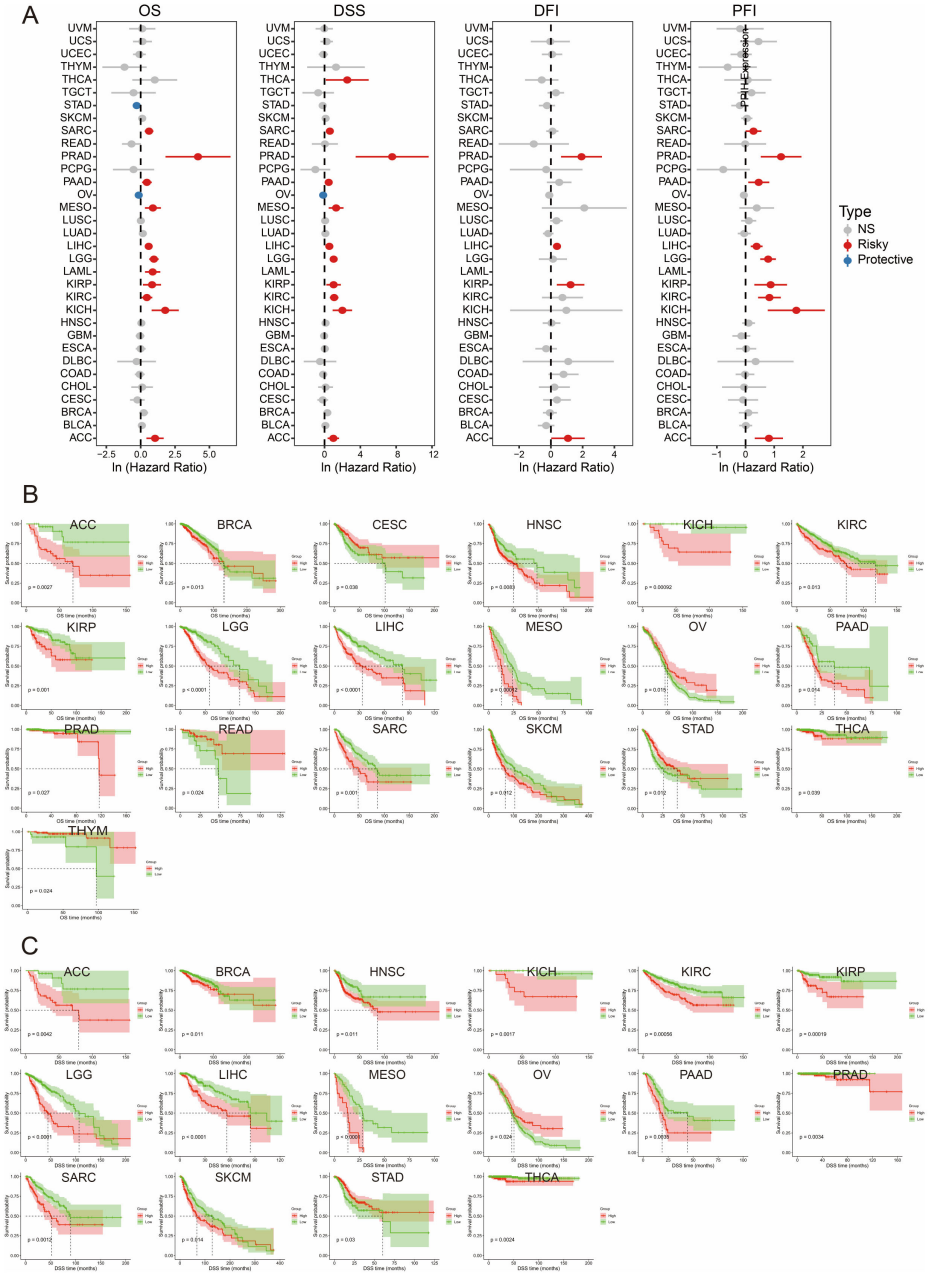
Pan-cancer survival analysis of PPIH. **(A)** Univariate Cox regression analysis of PPIH for OS, DSS, DFI and PFI. **(B, C)** Kaplan-Meier curves showing the differences in OS and DSS between patients with high and low PPIH expressions.

We first performed univariate Cox regression analyses to investigate the association between PPIH expression and various survival outcomes, including overall survival (OS), disease-specific survival (DSS), disease-free interval (DFI), and progression-free interval (PFI), across multiple malignancies. As shown in **Figure 3A**, elevated PPIH expression was predominantly associated with poor prognosis, acting as a potential risk factor in numerous cancer types. Notably, high PPIH expression was significantly correlated with shorter OS, DSS, and PFI in pancreatic adenocarcinoma. Similarly, negative correlations were observed between PPIH expression and all four survival indicators in liver hepatocellular carcinoma (LIHC), adrenocortical carcinoma (ACC), and prostate adenocarcinoma (PRAD). Interestingly, PPIH overexpression was paradoxically associated with favorable prognosis in stomach adenocarcinoma (STAD) and ovarian serous cystadenocarcinoma (OV), suggesting a potential context-dependent or stage-specific dual role of PPIH in tumor progression (18, 19). Subsequent Kaplan–Meier survival analyses further illustrated the clinical impact of PPIH expression. In terms of OS and DSS, high PPIH expression predicted significantly worse outcomes in multiple cancers, including LIHC, ACC, and breast invasive carcinoma (BRCA), while indicating improved survival in OV and select other cancer types (**Figures 3B, C**). Given the complex nature of cancer prognosis, we further explored the effect of PPIH dysregulation on DFI and PFI. The results demonstrated that PPIH expression may also significantly influence DFI and PFI in several tumor types (**Supplementary Figures S2A, B**). Additional Kaplan–Meier analyses for other cancer types are provided in **Supplementary Figure S3**. The survival analysis in this study highlights the clinical significance of PPIH as a prognostic biomarker across various cancer types. In multiple malignancies, elevated PPIH expression is closely associated with unfavorable survival outcomes, suggesting its potential involvement in promoting tumor progression, therapeutic resistance, or immune evasion. Conversely, in certain cancers, high PPIH expression is linked to improved prognosis, indicating a possible dual role that may depend on tumor subtype-specific contexts.

### 3.4 Significant heterogeneity of PPIH in the tumor microenvironment of hepatocellular carcinoma

**Figure 4:**
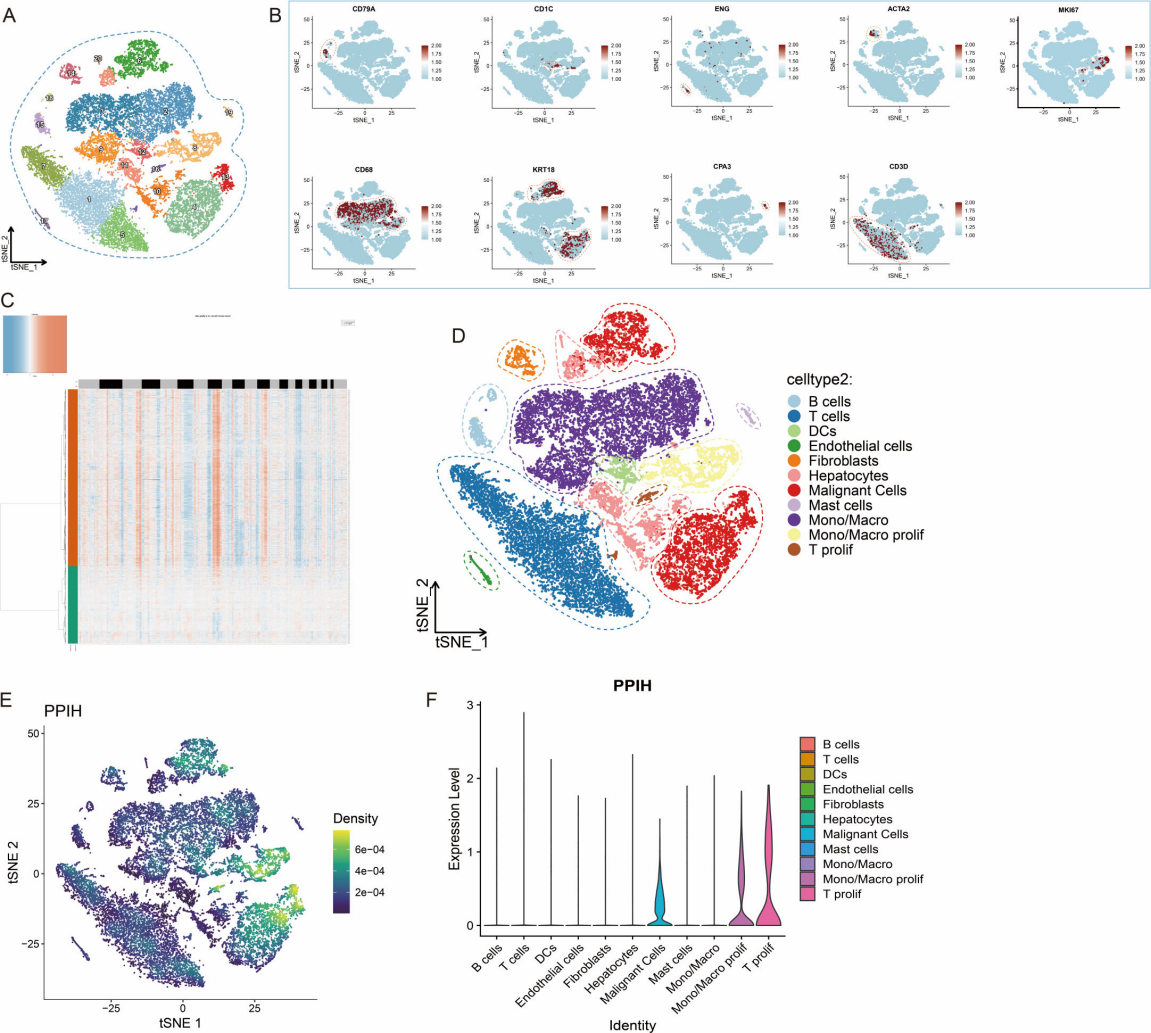
Heterogeneous Expression of PPIH in the Tumor Microenvironment of Hepatocellular Carcinoma. **(A)** t-SNE plot showing distinct cell clusters in HCC. **(B)** Feature plots illustrating cell type–specific marker genes in HCC tissues. **(C)** Identification of malignant cells in HCC samples using CopyKAT. **(D)** t-SNE plot displaying the major cell types within the HCC tumor microenvironment. **(E)** Feature plots showing the distribution of PPIH expression across different cell types in HCC tissues. **(F)** Violin plot demonstrating the prominent enrichment of PPIH expression in malignant cells, proliferative monocytes/macrophages, and proliferative T cells.

To further investigate the heterogeneity of PPIH expression within the tumor microenvironment of HCC, we analyzed single-cell RNA sequencing (scRNA-seq) data from the GEO database (GSE166635). All samples were derived from patients diagnosed with HCC. Following stringent quality control procedures, 29,000 high-quality cells were retained for downstream analysis. We performed principal component analysis (PCA) and t-distributed stochastic neighbor embedding (t-SNE) for dimensionality reduction and clustering, resulting in the identification of 20 distinct cellular clusters (**Figure 4A**). Cell types were annotated using well-established marker genes, including CD3D for T cells, CD79A for B cells, CD68 for monocytes and macrophages, CD1C for dendritic cells, ENG for endothelial cells, ACTA2 for fibroblasts, KRT18 for epithelial cells, CPA3 for mast cells, and MKI67 for proliferating cells (**Figure 4B**). To identify malignant cells, we applied the Bayesian segmentation algorithm from CopyKAT, which infers genome-wide copy number variation (CNV) profiles from scRNA-seq data at a resolution of approximately 5 Mb. Based on CNV patterns, epithelial cells were classified as either aneuploid (malignant) or diploid (non-malignant) (**Figure 4C**), and the full dataset was ultimately annotated into 11 major cell types (**Figure 4D**). PPIH expression demonstrated pronounced intercellular heterogeneity, with particularly high levels observed in malignant epithelial cells and proliferative monocyte/macrophage subsets (**Figure 4E**). This pattern was further validated by violin plots showing distinct, cell type-specific expression profiles of PPIH (**Figure 4F**). The variable expression of PPIH within the HCC microenvironment carries potential biological significance. First, its preferential enrichment in tumor cells suggests a possible involvement in tumor cell proliferation or maintenance, consistent with its previously reported oncogenic roles. Second, its elevated expression in proliferating monocytes and macrophages raises the possibility that PPIH may be involved in modulating tumor-associated immune responses. Together, these findings underscore the complexity of PPIH regulation in HCC and highlight its potential relevance in both tumor cell-intrinsic and microenvironmental contexts.

### 3.5 PPIH Promotes malignant proliferation of tumor cells and modulates the tumor microenvironment

**Figure 5:**
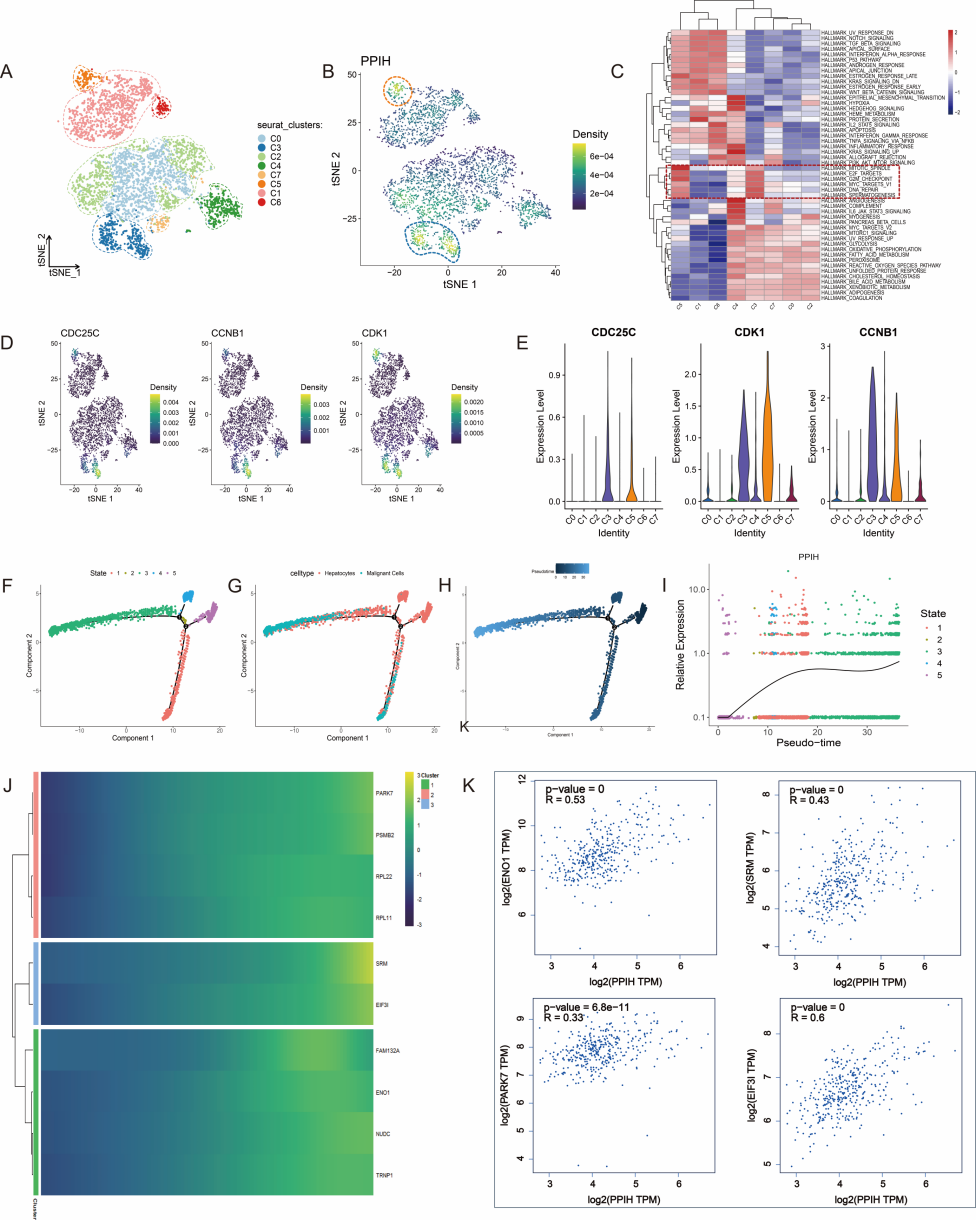
PPIH overexpression significantly promotes malignant proliferation of tumor cells and may contribute to hepatocyte transformation. **(A)** Distinct subpopulations of tumor cells. **(B)** Enrichment of PPIH expression in the C3 and C5 subclusters of tumor cells. **(C)** Tumor cells in the C3 and C5 subclusters exhibit a higher proliferative tendency compared to other tumor cell populations. **(D, E)** The expression patterns of CDC25C, CCNB1, and CDK1 in malignant cells were visualized using the Nebulosa package and violin plots. **(F–H)** Pseudotime analysis reveals the differentiation trajectory from hepatocytes to malignant cells. **(I)** PPIH expression gradually increases along the differentiation axis from hepatocytes to malignant cells. **(J)** Cluster heatmap showing genes highly correlated with pseudotime trajectory nodes. **(K)** PPIH expression is significantly positively correlated with multiple node-associated genes.

To dissect the functional heterogeneity of PPIH in malignant cells, we stratified tumor cells into eight subclusters (**Figure 5A**). Nuclear density analysis using the Nebulosa package revealed significant PPIH enrichment in subclusters C3 and C5 (**Figure 5B**). Subsequent Gene Set Variation Analysis (GSVA) indicated stronger proliferative activity in these subclusters, with C3/C5 exhibiting higher scores in cancer-related pathways—including E2F targets, G2M checkpoint, and MYC targets V2—compared to other subclusters (**Figure 5C**). These results suggest that PPIH is a key regulator of tumor cell proliferation and may drive HCC progression. Meanwhile, we observed a significant overlap in the expression patterns of key cell cycle regulators, including CDC25C, CCNB1, and CDK1, with PPIH in malignant cells (**Figure 5D–E**), further suggesting that PPIH may promote tumor cell proliferation by modulating cell cycle progression. Pseudotime trajectory analysis based on Monocle2 reconstructed a continuous differentiation process from hepatocytes to malignant cells, identifying five distinct states. According to two branching points, cellular states were defined; with advancing pseudotime, cells progressively differentiated from states 4 and 5 to states 1 and 3 (**Figure 5F**). Visualization of this trajectory confirmed progressive malignant transformation (**Figures 5G, H**). Notably, PPIH expression showed pseudotime-dependent upregulation during hepatocyte-to-malignant cell transition (**Figure 5I**). Branch point analysis identified key regulators of this process (**Figure 5J**), and Spearman correlation analysis revealed significant positive associations between PPIH and multiple branch-defining genes (**Figure 5K**). These multilayered analyses demonstrate that PPIH serves not only as a biomarker but is also potentially involved in the process of hepatic malignant transformation, although further mechanistic validation is warranted.

To investigate how PPIH overexpression remodels the TME, we performed cell-cell communication analysis using CellChat, focusing on ligand-receptor (LR) interactions mediated by PPIH-enriched malignant cells and proliferative monocyte/macrophage subsets. Bubble plots visualized the directional signaling roles of 11 cellular subtypes in outgoing and incoming pathways (**Supplementary Figures S4A, B**). Malignant cells acted as primary signaling sources for midkine (MK), targeting diverse immune and stromal populations. Proliferative monocytes/macrophages predominantly activated the secreted phosphoprotein 1 (SPP1) pathway. Quantitative circle diagrams delineated interaction network topology and signaling strength across cell types (**Supplementary Figures S4C, D**). Pathway decomposition revealed selective targeting of stromal components by both MK and SPP1 signaling pathways (**Supplementary Figures S4E, F**). Detailed LR mapping identified ITGB1 as the core receptor enabling malignant cells to regulate fibroblasts and endothelial cells (**Supplementary Figure S4G**)—a mechanism associated with epithelial-mesenchymal transition (EMT) and poor prognosis in multiple cancers (20, 21). Similarly, proliferative monocytes/macrophages engaged stromal cells via ITGB1 while activating mast cells through SPP1-CD44 interactions (**Supplementary Figure S4H**), which promote tumor stemness and immunosuppression (22–24). These findings delineate a dual mechanistic framework: 1) PPIH-enriched malignant cells may potentiate EMT through MK/ITGB1-mediated stromal reprogramming, and 2) cycling myeloid populations could reinforce immunosuppression via SPP1-CD44 network. Collectively, these pathway-specific perturbations provide a plausible explanation for PPIH-associated TME dysregulation, contributing to unfavorable clinical outcomes in HCC. Through single-cell analyses, we identified a potential role of PPIH in malignant cell proliferation. The pseudotime analysis suggests that further studies are necessary to validate the relationship between PPIH and hepatocyte malignant transformation. Additionally, cell-cell communication analysis reveals dual roles of PPIH-high malignant cells and proliferative myeloid cells in remodeling the tumor microenvironment. Collectively, these findings elucidate the important functions of PPIH in promoting hepatocellular carcinoma progression and modulating the tumor microenvironment.

### 3.6 PPIH in tumor immunity and genomic alterations

**Figure 6:**
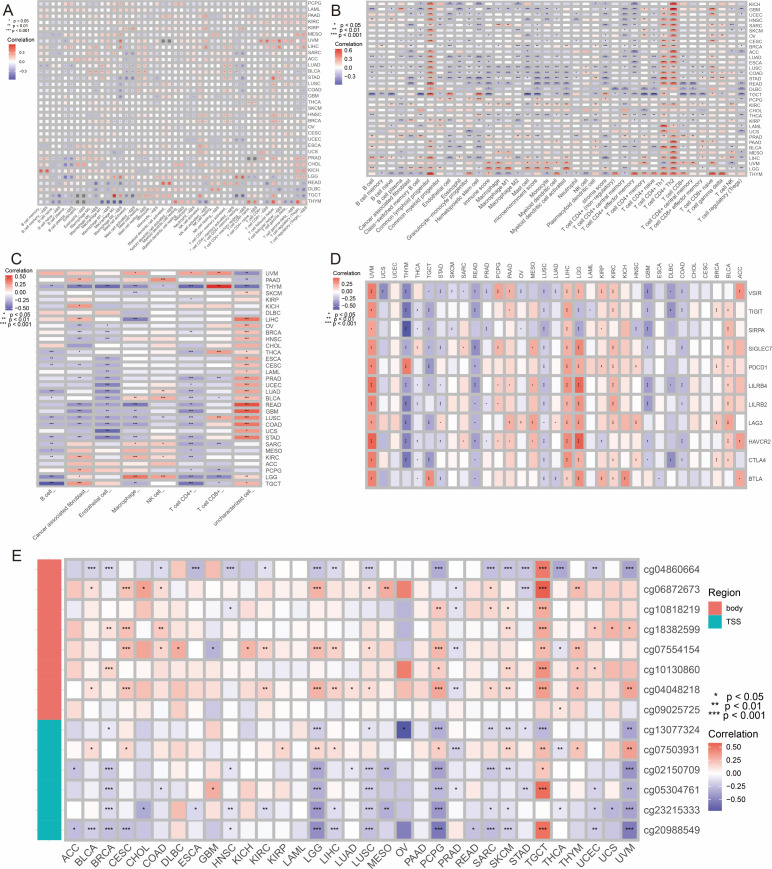
Immune and epigenetic associations of PPIH in pan-cancer. **(A–C)** Correlations of PPIH expressions with immune cell infiltraion levels quantified by XCELL, CIBERSORT-ABS and EPIC algorithms. **(D)** Expression correlations of PPIH and various immune checkpoint genes. **(E)** Correlations of PPIH expressions with its methylation levels at different CpG sites within the promotor region.

We performed a comprehensive multi-omics analysis to elucidate the potential role of PPIH in cancer immunity and tumorigenesis. Immune infiltration analysis based on XCELL, CIBERSORT-ABS, and EPIC algorithms revealed that PPIH expression was significantly correlated with the infiltration levels of various immune cell subsets across cancer types (**Figures 6A–C**). Notably, strong associations were observed between PPIH expression and lymphocyte populations such as Th1, Th2 (XCELL), and natural killer (NK) cells (EPIC), suggesting a key role for PPIH in shaping the tumor immune microenvironment (TME). In parallel, PPIH expression was also significantly correlated with multiple immune checkpoint molecules, implying its involvement in immune escape mechanisms and its potential as a predictive biomarker for immunotherapeutic responsiveness (**Figure 6D**).

Epigenetically, we identified DNA methylation as a critical regulatory mechanism governing PPIH transcription. Integration of expression and methylation data demonstrated a significant negative correlation between PPIH mRNA levels and promoter methylation at multiple CpG sites (**Figure 6E**), implicating promoter hypomethylation in PPIH overexpression. UALCAN-based pan-cancer analysis further confirmed that tumor tissues in several malignancies—including BLCA, HNSC, LUAD, PRAD, READ, TGCT, THCA, and UCEC—exhibited significantly lower methylation levels compared to normal controls (**Supplementary Figure S5B**). Interestingly, PPIH methylation was also strongly associated with both immune cell infiltration and immune checkpoint gene expression, indicating its potential dual role in tumor progression and immune modulation (**Supplementary Figure S5A**). On the genomic level, PPIH alterations were profiled across 33 cancer types using the cBioPortal platform. Gene amplification emerged as the dominant alteration in OV, ESCA, and BRCA, while SKCM exhibited a mixed pattern of mutations and amplifications (**Supplementary Figure S6A**). Mutation frequency was notably elevated in UCEC and CESC (**Supplementary Figure S6B**), and survival analysis showed that patients with PPIH mutations had significantly worse overall survival compared to those without (**Supplementary Figure S6C**). Structural mapping indicated that mutation hotspots were mainly located within the functional peptidyl-prolyl isomerase domain (**Supplementary Figure S6D**). Furthermore, CNV analysis revealed that increased PPIH copy number was positively correlated with immunoregulatory genes, including immune checkpoints, MHC molecules, and immunosuppressive cytokines in KICH, LGG, and UVM (**Supplementary Figure S6E**), reinforcing the notion that genetic alterations in PPIH contribute to TME remodeling. Overall, multi-omics analyses demonstrate that PPIH plays a pivotal role in cancer immune regulation and tumor progression. Its expression is significantly correlated with the infiltration of various immune cells and immune checkpoint molecules, highlighting its involvement in modulating the tumor immune microenvironment. Promoter hypomethylation and frequent genomic alterations further support its role in tumorigenesis and immune evasion. These findings suggest that PPIH may serve as an important biomarker and therapeutic target for cancer immunotherapy.

### 3.7 Functional enrichment analysis of PPIH

**Figure 7:**
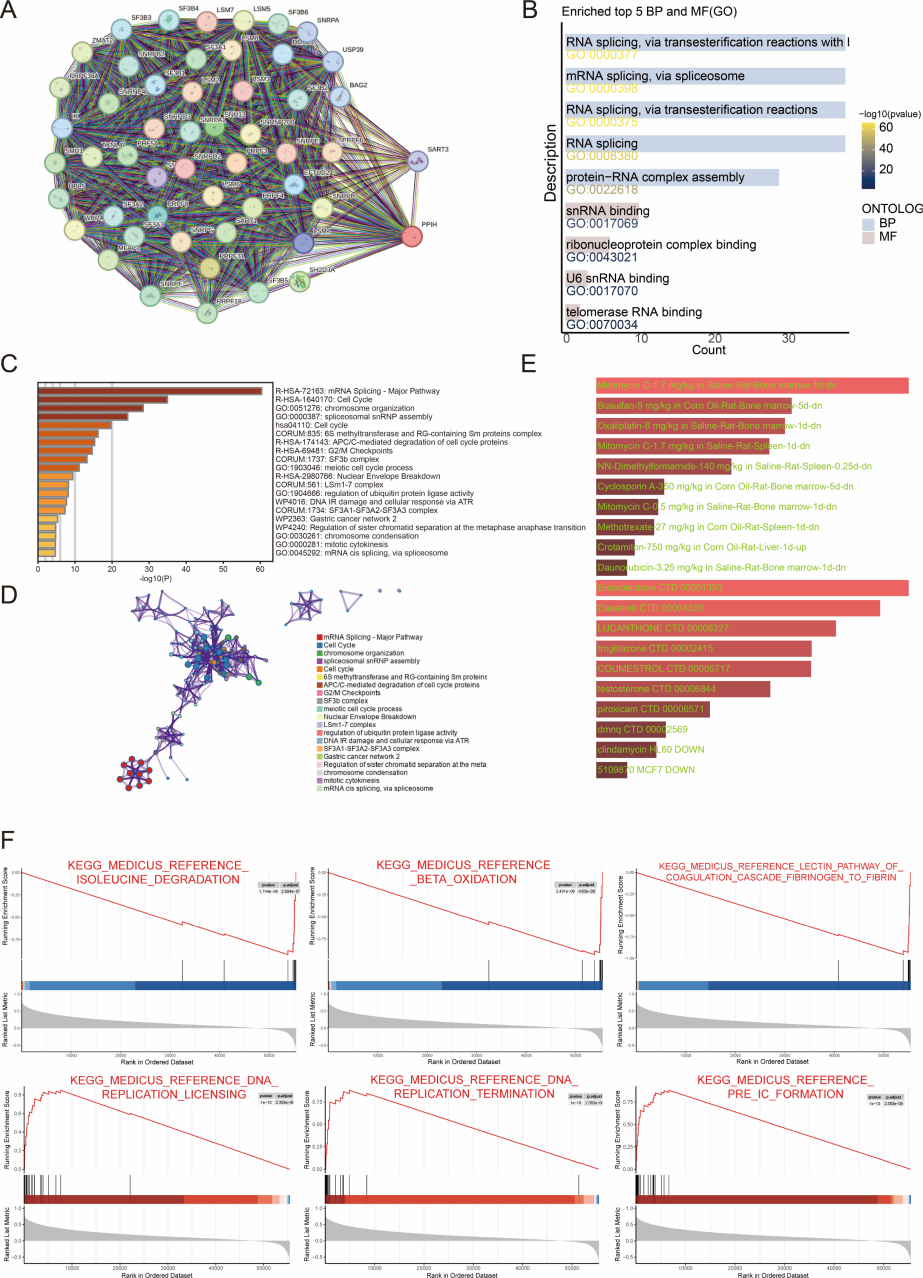
Functional enrichment analysis of PPIH. **(A)** The PPI network of PPIH. **(B, C)** Enrichment analysis of PPIH’s interactive proteins. **(D)** MetaScape enrichment analysis of PPIH’s interactive proteins. **(E)** The potential drug targets of PPIH predicted by DsigDB and DrugMatrix. **(F)** GSEA analysis of PPIH.

To gain insights into the potential molecular functions of PPIH in tumor progression, we first constructed its protein–protein interaction (PPI) network using the STRING database. This approach yielded a set of proteins with putative functional associations with PPIH (**Figure 7A**). Subsequent functional enrichment analysis of these candidate genes, performed using Gene Ontology (GO) and Metascape, revealed that they are significantly involved in biological processes such as RNA splicing, DNA replication, and cell cycle regulation (**Figures 7B–D**). To further explore the biological relevance of PPIH expression in HCC, we conducted gene set enrichment analysis (GSEA) based on HCC transcriptomic profiles. The results revealed a clear functional divergence associated with PPIH expression levels: low PPIH expression was predominantly correlated with enrichment of hepatic metabolic pathways, while high PPIH expression was significantly associated with activation of DNA replication and cell cycle–related pathways (**Figure 7F**). In addition, we explored the therapeutic implications of PPIH by predicting potential drug interactions. Using DSigDB and DrugMatrix, we identified candidate compounds targeting PPIH and its highly co-expressed genes (**Figure 7E**). These findings suggest that PPIH may contribute to poor cancer prognosis by promoting malignant proliferation of tumor cells through the regulation of cell cycle–related pathways.

### 3.8 PPIH was upregulated in HCC

**Figure 8:**
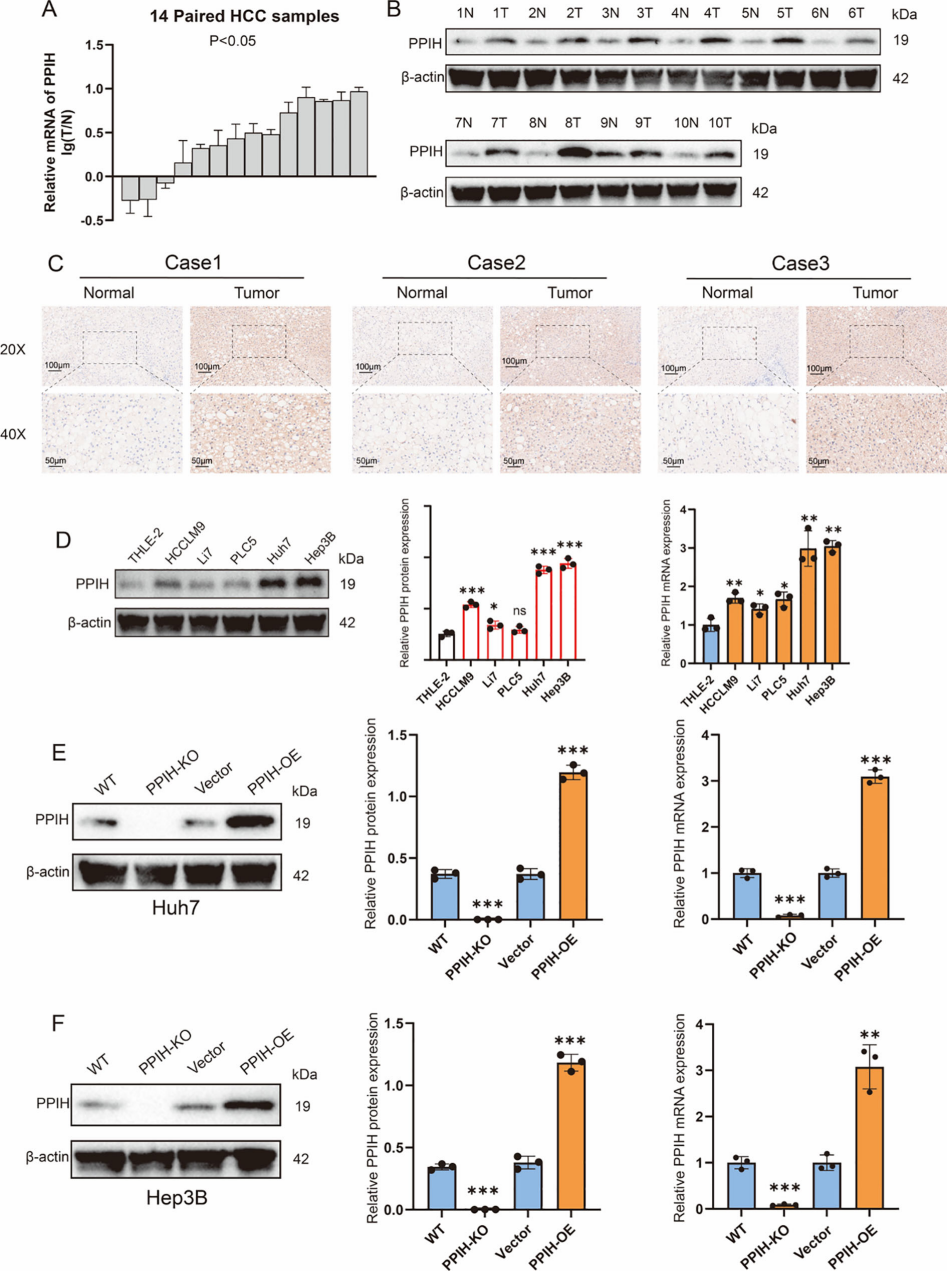
PPIH is upregulated in HCC. **(A)** Relative mRNA expression of PPIH in normal liver tissues and HCC tissues. **(B)** Western blot analysis of PPIH protein levels in normal liver and HCC tissues. **(C)** Immunohistochemical staining showing PPIH protein expression in normal liver and HCC tissues. **(D)** Expression of PPIH in immortalized hepatocytes and HCC cell lines. **(E)** Knockout and overexpression of PPIH in Huh7 cells. **(F)** Knockout and overexpression of PPIH in Hep3B cells. *p < 0.05; **p < 0.01 and ***p < 0.001.

To validate the results from public database analyses, we assessed PPIH expression in hepatocellular carcinoma (HCC). qRT-PCR analysis of 14 randomly selected paired HCC tissues and adjacent normal liver tissues from the sample database of the Second Hospital of Shandong University revealed that the transcriptional level of PPIH was significantly upregulated in HCC tissues (**Figure 8A**). This upregulation at the protein level was further confirmed by Western blotting and immunohistochemistry (**Figures 8B, C**). Consistently, both mRNA and protein levels of PPIH were elevated in four HCC cell lines compared to the normal human hepatic cell line THLE-2, with especially pronounced expression in Huh7 and Hep3B cells (**Figure 8D**). Subsequently, we established PPIH knockout and overexpression cell lines and validated PPIH expression at both mRNA and protein levels (**Figures 8E, F**).

### 3.9 Overexpression of PPIH enhances the proliferation, invasion, and migration of hepatocellular carcinoma cells

**Figure 9:**
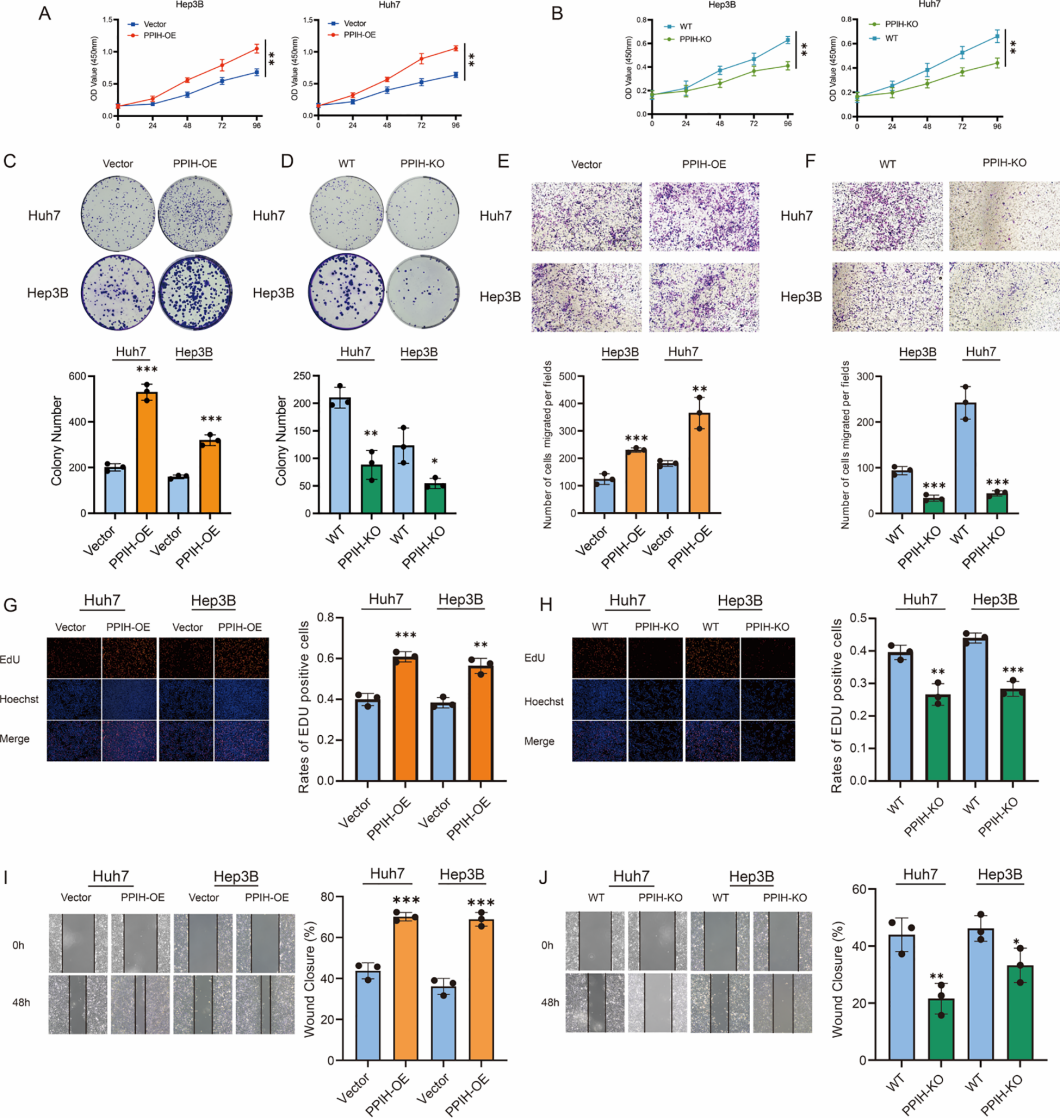
Functional effects of PPIH on hepatocellular carcinoma cells. **(A, B)** CCK-8 assays revealed that PPIH overexpression or knockout significantly affected HCC cell proliferation. **(C, D)** Colony formation assays showed significant differences in clonogenic potential between the PPIH-overexpression or knockout groups and the corresponding control (Vector or WT) groups. **(E, F)** Transwell migration assays demonstrated that PPIH overexpression enhanced, while knockout suppressed, the migratory capability of HCC cells. **(G, H)** EdU assays indicated that PPIH overexpression and knockout significantly influenced the proliferative activity of HCC cells. **(I, J)** Wound healing assays showed that PPIH overexpression accelerated, while knockout impaired, cell migration dynamics over a 48-hour period. Data are presented as mean ± SEM from three independent experiments. Statistical significance was assessed by two-tailed Student’s t-test: ***p < 0.001, **p < 0.01, * *p < 0.05*.

To investigate the biological role of PPIH in HCC, we systematically evaluated the effects of PPIH on the proliferation, invasion, and migration of Huh7 and Hep3B cells through a series of *in vitro* experiments. CCK-8 assays revealed that stable overexpression of PPIH significantly promoted the proliferation of HCC cells (**Figure 9A**), while PPIH knockout markedly suppressed cell viability (**Figure 9B**). Colony formation assays further confirmed that PPIH overexpression significantly enhanced the colony-forming ability of both Huh7 and Hep3B cells, whereas PPIH knockout substantially reduced this capacity (**Figures 9C, D**). To further explore the effect of PPIH on the migratory capacities of HCC cells, Transwell and wound healing assays were performed. Transwell assays showed that PPIH overexpression markedly increased the migratory potential of Huh7 and Hep3B cells, while PPIH knockout significantly suppressed their migration (**Figures 9E, F**). EdU incorporation assays demonstrated that PPIH overexpression significantly increased the proliferation index of HCC cells, whereas PPIH knockout led to a decrease in proliferative activity (**Figures 9G, H**). Consistent with these findings, wound healing assays revealed that PPIH overexpression promoted cell migration, while knockout of PPIH significantly impaired the migratory ability of HCC cells (**Figures 9I, J**). Statistical analysis indicated that PPIH overexpression significantly enhanced tumor-associated biological behaviors in all functional assays, whereas PPIH knockout led to a marked reduction in cellular activity (*p < 0.05*).

## 4 Discussion

In recent years, bioinformatics approaches such as MR have emerged as powerful tools for identifying aberrant genomic alterations involved in carcinogenesis. However, traditional MR methods face limitations in establishing causal relationships due to confounding factors and reverse causation. Against this backdrop, SMR has been developed as a more robust analytical framework. SMR leverages genetic variants as instrumental variables to infer causal associations between exposure biomarkers and disease outcomes while effectively minimizing environmental confounding (25, 26). Given the advantages of SMR, we employed this approach to systematically screen for potential cancer driver genes. Among the three malignant tumor GWAS datasets included, PPIH was identified as a common critical gene across different cancer types and was therefore selected as a candidate target for further in-depth investigation.

PPIH, a member of the cyclophilin family, catalyzes the cis-trans isomerization of proline residues, inducing local conformational changes in surrounding protein structures (27). Emerging evidence indicates that PPIH is closely implicated in the pathogenesis of multiple malignancies, with notably elevated expression observed in LIHC and COAD (28). Furthermore, PPIH expression correlates significantly with immune cell infiltration in HCC, gastric cancer (GC), and CHOL, suggesting that its pro-tumorigenic effects may be mediated via modulation of the TME (29–32). Our study corroborates these findings by demonstrating that PPIH is overexpressed in multiple cancer types and is associated with poor clinical outcomes, thereby strengthening its credibility as a pan-cancer biomarker. Notably, prior studies have primarily focused on the context-dependent functions of PPIH within individual cancer subtypes, often relying on single-cohort analyses with limited sample sizes and lacking systematic evaluation. Consequently, the broader biological relevance of PPIH across diverse malignancies remains to be fully elucidated, particularly regarding its epigenetic regulation, biological functions, and interactions with TME components.

To address these gaps, we first conducted a comprehensive analysis of PPIH’s genetic alteration landscape across multiple cancers, including somatic mutations, copy number variations (CNVs), and DNA methylation patterns. Our results indicate that PPIH overexpression is largely attributable to its high mutation frequency, copy number amplification, and promoter hypomethylation. Subsequent functional enrichment analyses revealed that PPIH is primarily involved in RNA splicing, DNA replication, and cell cycle regulation. It is worth mentioning that previous studies have reported that precursor RNAs of numerous oncogenes require selective splicing to generate mature mRNA transcripts with oncogenic activity (33–35). Moreover, the significant enrichment of DNA replication and cell cycle-related pathways further suggests that PPIH may promote tumor cell proliferation by facilitating cell cycle progression. Collectively, these findings provide novel insights into the molecular mechanisms underlying PPIH-mediated tumorigenesis and offer new directions for future mechanistic investigations.

In addition to cancer cells, the TME comprises diverse cellular components such as immune and stromal cells, as well as non-cellular elements including vascular structures, ECM, and a variety of signaling molecules. This dynamic and highly heterogeneous ecosystem plays a pivotal role in malignant progression (36). Utilizing multiple computational algorithms, our study demonstrated a significant correlation between PPIH expression and infiltration of various immune cell types. Integration with hepatocellular carcinoma single-cell RNA sequencing data further elucidated the potential regulatory role of PPIH within the TME. Key findings include: (1) marked enrichment of PPIH in malignant cells and proliferative monocyte/macrophage subclusters; (2) enhanced proliferative capacity of high PPIH-expressing malignant cell subpopulations relative to others; (3) pseudotime trajectory analysis revealing a progressive increase of PPIH expression during hepatocyte malignant transformation, implying its involvement in driving malignant evolution; (4) cell–cell communication analysis identifying MK and SPP1 signaling pathways as major mediators between PPIH-high malignant/proliferative monocyte/macrophage cells and other TME constituents. The oncogenic roles of these pathways have been previously validated in various cancers (37–39), thereby reinforcing the credibility of our single-cell findings. Importantly, *in vitro* experiments confirmed significant upregulation of PPIH in HCC tissues, with overexpression markedly enhancing HCC cell proliferation, invasion, and migration, whereas PPIH knockdown suppressed these malignant phenotypes, supporting its oncogenic function in HCC.

Admittedly, our study has several limitations. First, despite leveraging GWAS datasets of multi-cancers, we did not identify genetic variants significantly associated with PPIH protein expression. Second, the GWAS data predominantly derive from European ancestry populations, which may limit the generalizability of our findings to other ethnic groups. Finally, although preliminary *in vitro* assays verified the pro-tumorigenic role of PPIH, its precise molecular regulatory network remains to be fully characterized. In particular, future studies are warranted to dissect its interactions with key signaling molecules and clarify its functional role in TME remodeling.

## 6 Conclusions

In summary, this study first identified PPIH as a candidate biomarker of different system-derived malignancies through SMR analysis. Further multi-omics analyses confirmed the potential utility of PPIH as a pan-cancer diagnostic and prognostic biomarker gene. Importantly, we systematically elucidated the potential role of PPIH in tumorigenesis and revealed its functional correlation with the TME through scRNA-seq analysis. These findings deepen our understanding of PPIH’s multifaceted roles in cancer progression and provide novel insights for the research and development of targeted therapeutic strategies.

## Data Availability

The original contributions presented in the study are included in the article/**Supplementary Material**. Further inquiries can be directed to the corresponding author.
