# Supplementary figures and images for: eQTL and multi-omics integration reveal PPIH as a prognostic and immunotherapeutic biomarker
Source: Front Immunol. 2025 Aug 14;16:1647722. doi: 10.3389/fimmu.2025.1647722 (PMC12391104; doi:10.3389/fimmu.2025.1647722)

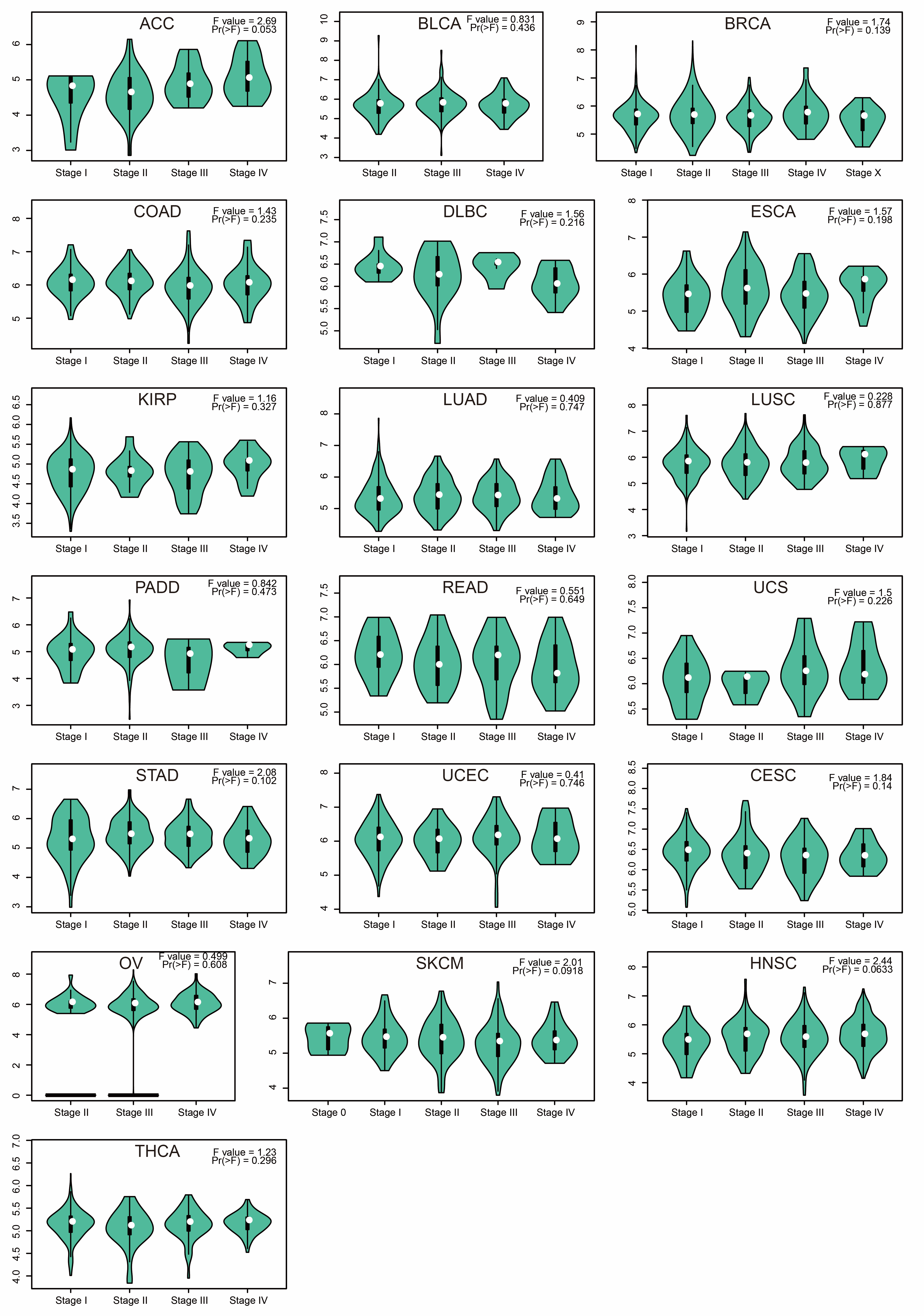

Supplement: Supplementary file 3 [file Image1.tif]

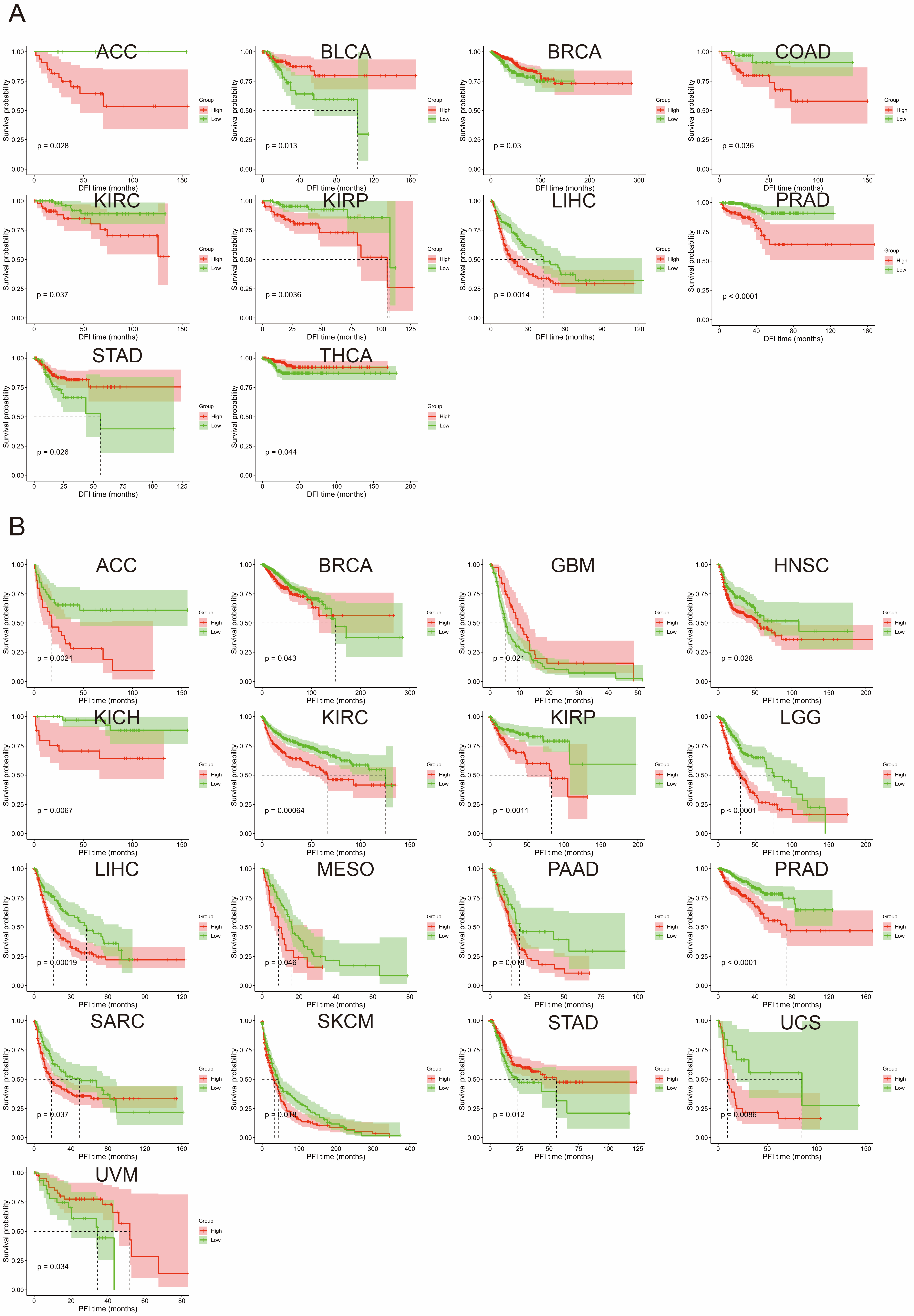

Supplement: Supplementary file 4 [file Image2.tif]

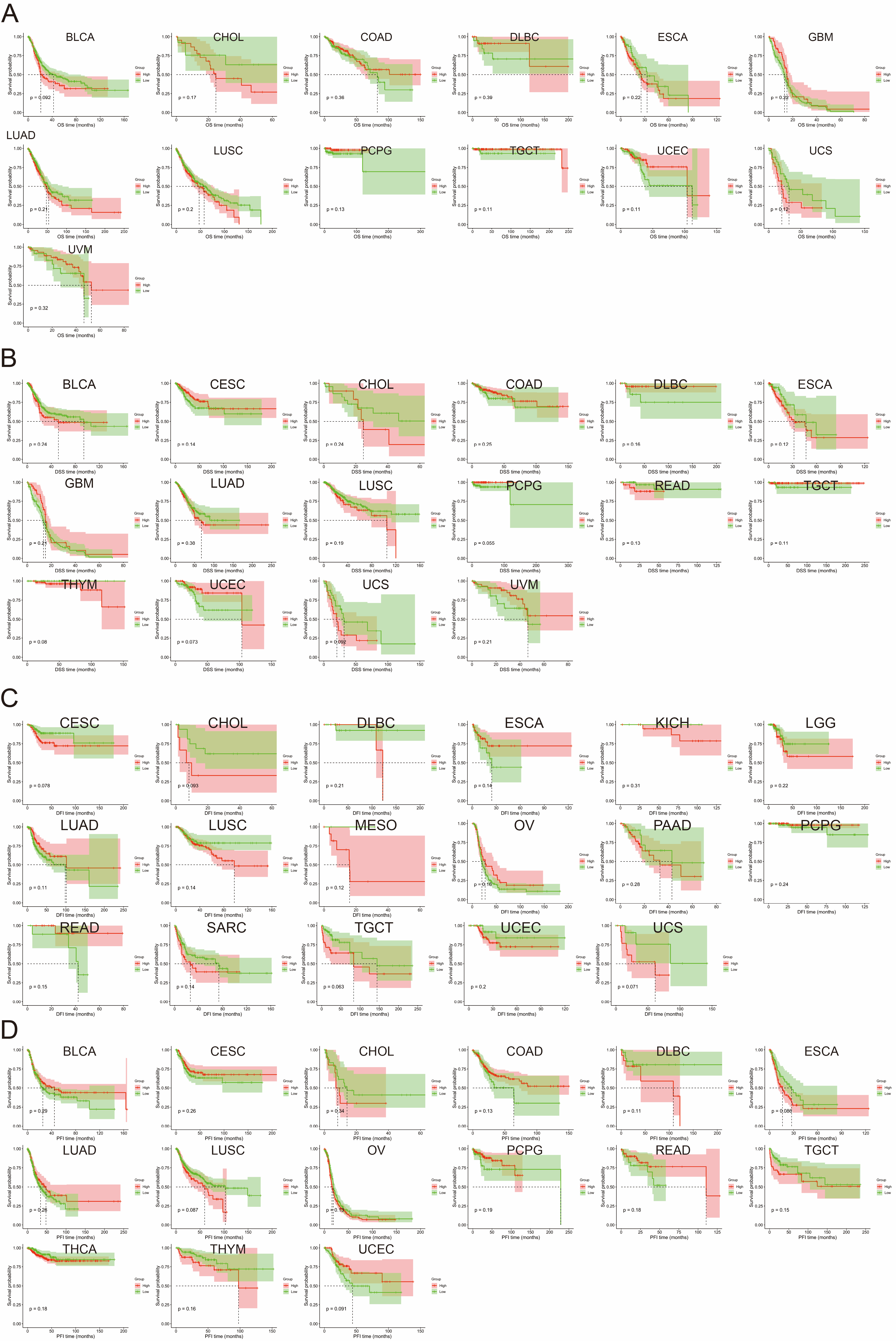

Supplement: Supplementary file 5 [file Image3.tif]

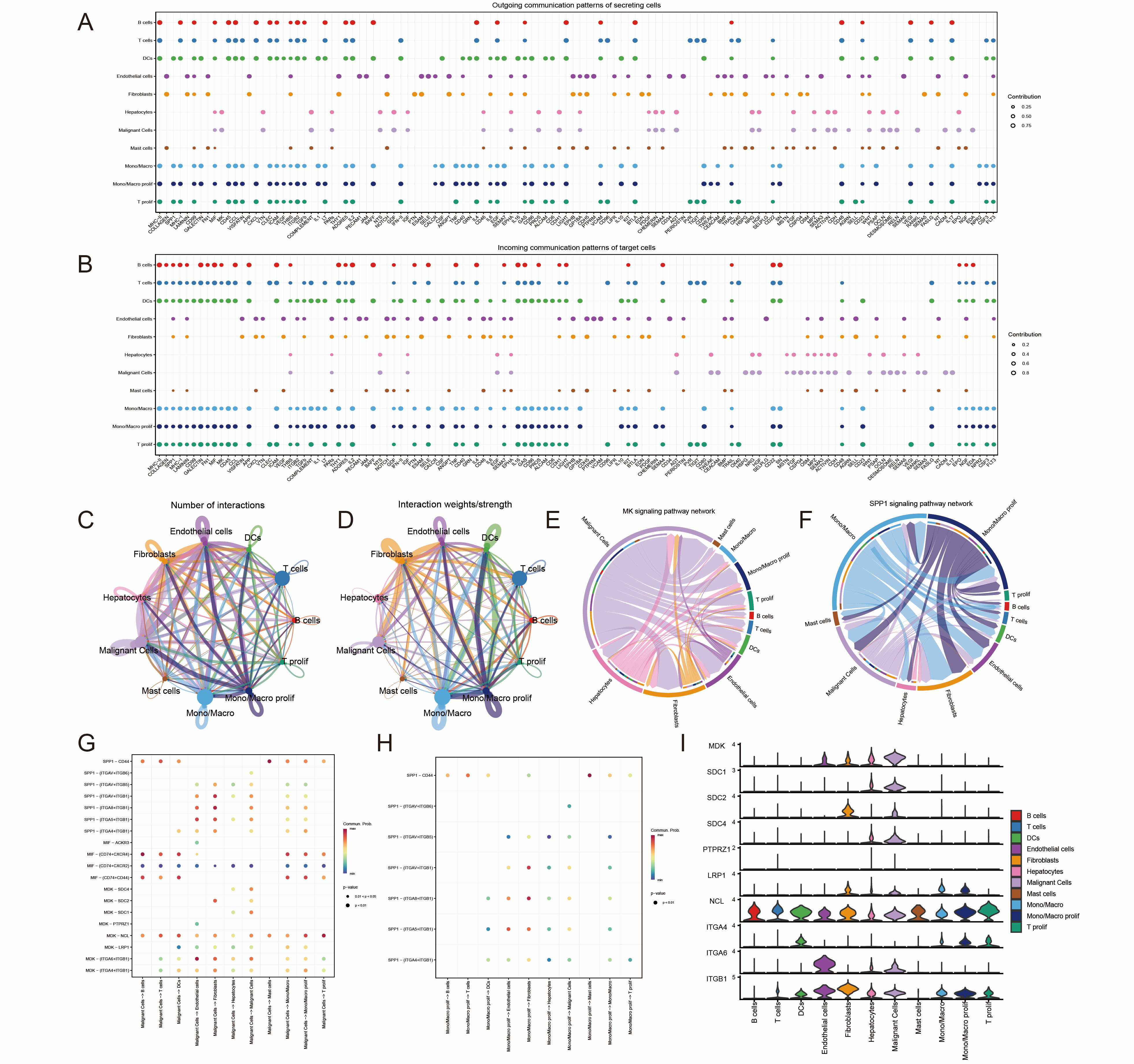

Supplement: Supplementary file 6 [file Image4.tif]

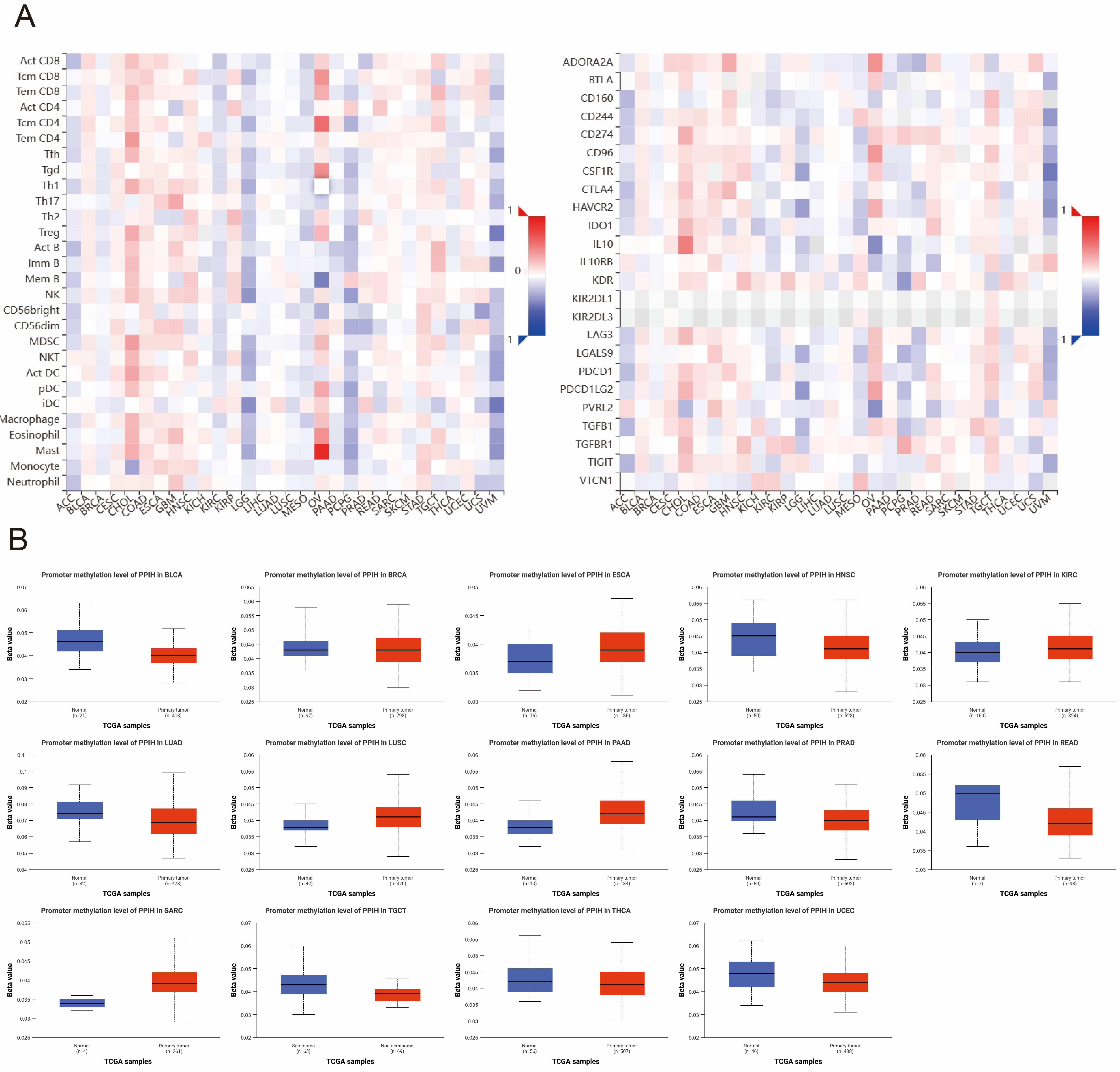

Supplement: Supplementary file 7 [file Image5.tif]
