# Supplementary material for: eQTL and multi-omics integration reveal PPIH as a prognostic and immunotherapeutic biomarker
Source: Front Immunol. 2025 Aug 14;16:1647722. doi: 10.3389/fimmu.2025.1647722 (PMC12391104; doi:10.3389/fimmu.2025.1647722)
Supplement: Supplementary file 8 [file Presentation1.pdf]

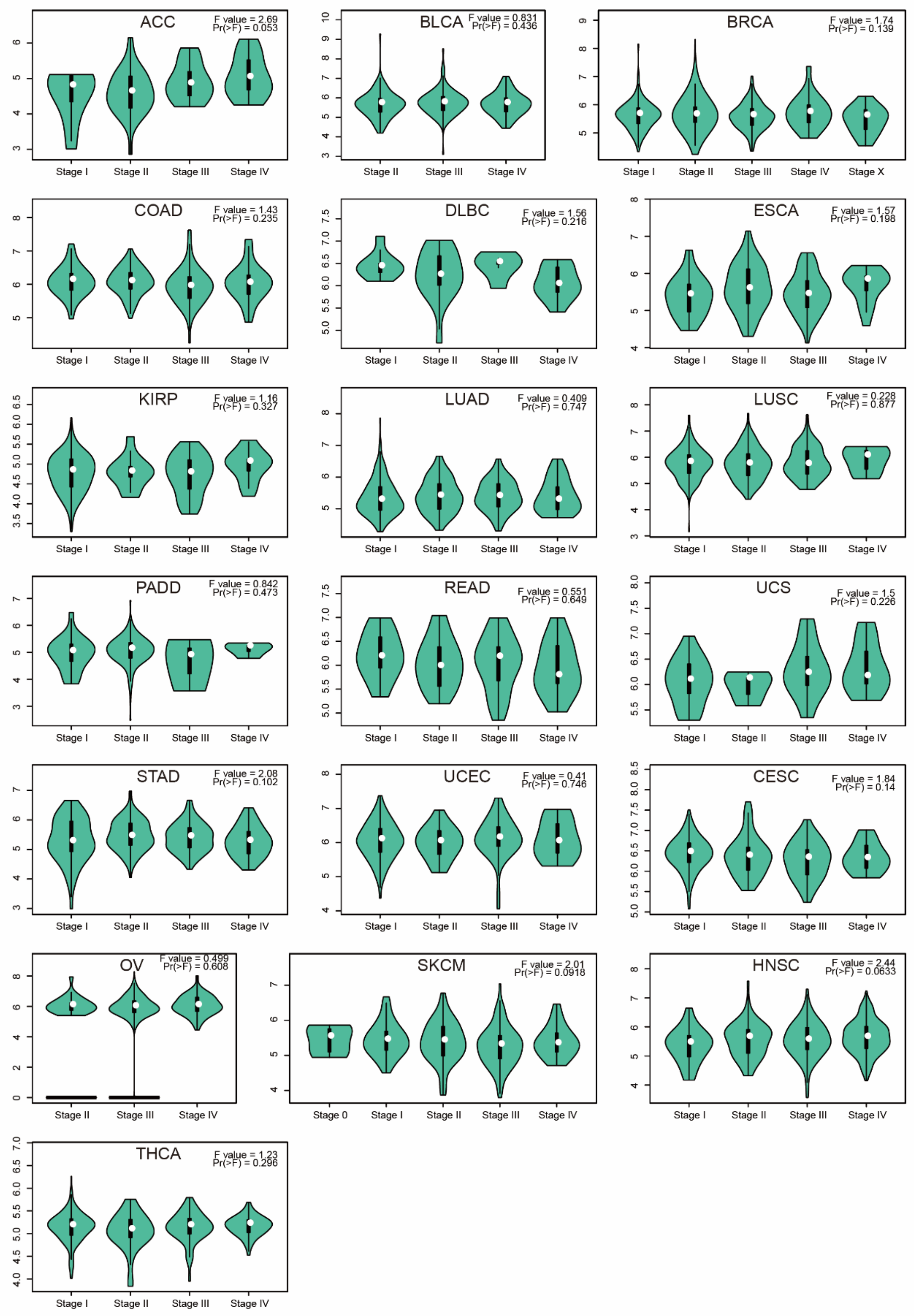

Figure S1. Other cancers with no significant correlation between clinical stage and PPIH expression.

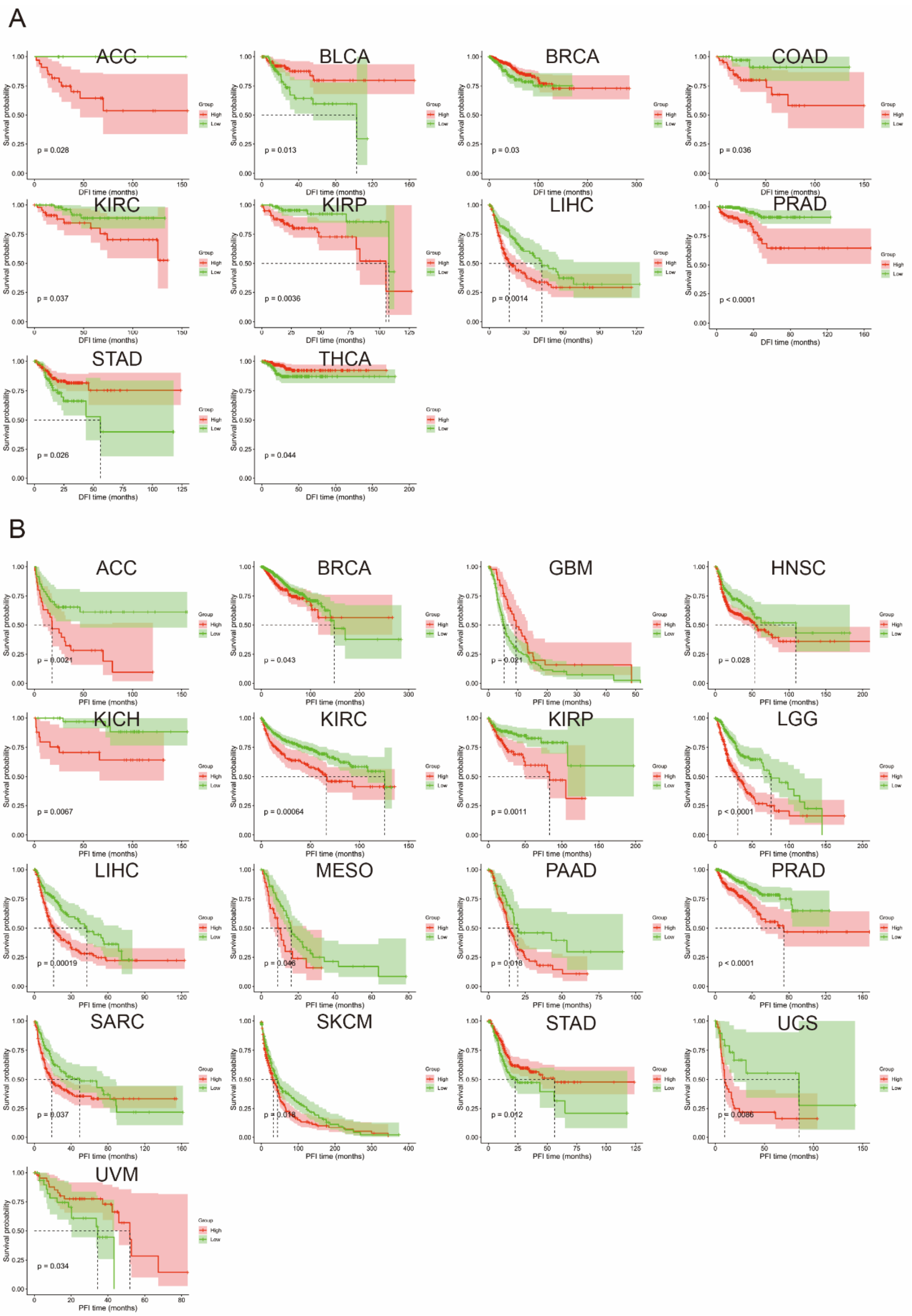

Figure S2. Impact of aberrant PPIH expression on DFI and PFI in cancer patients.

(A) Overexpression of PPIH significantly affects DFI in various cancer patients. **(B)** Aberrant expression of PPIH significantly influences PFI in multiple cancer types.

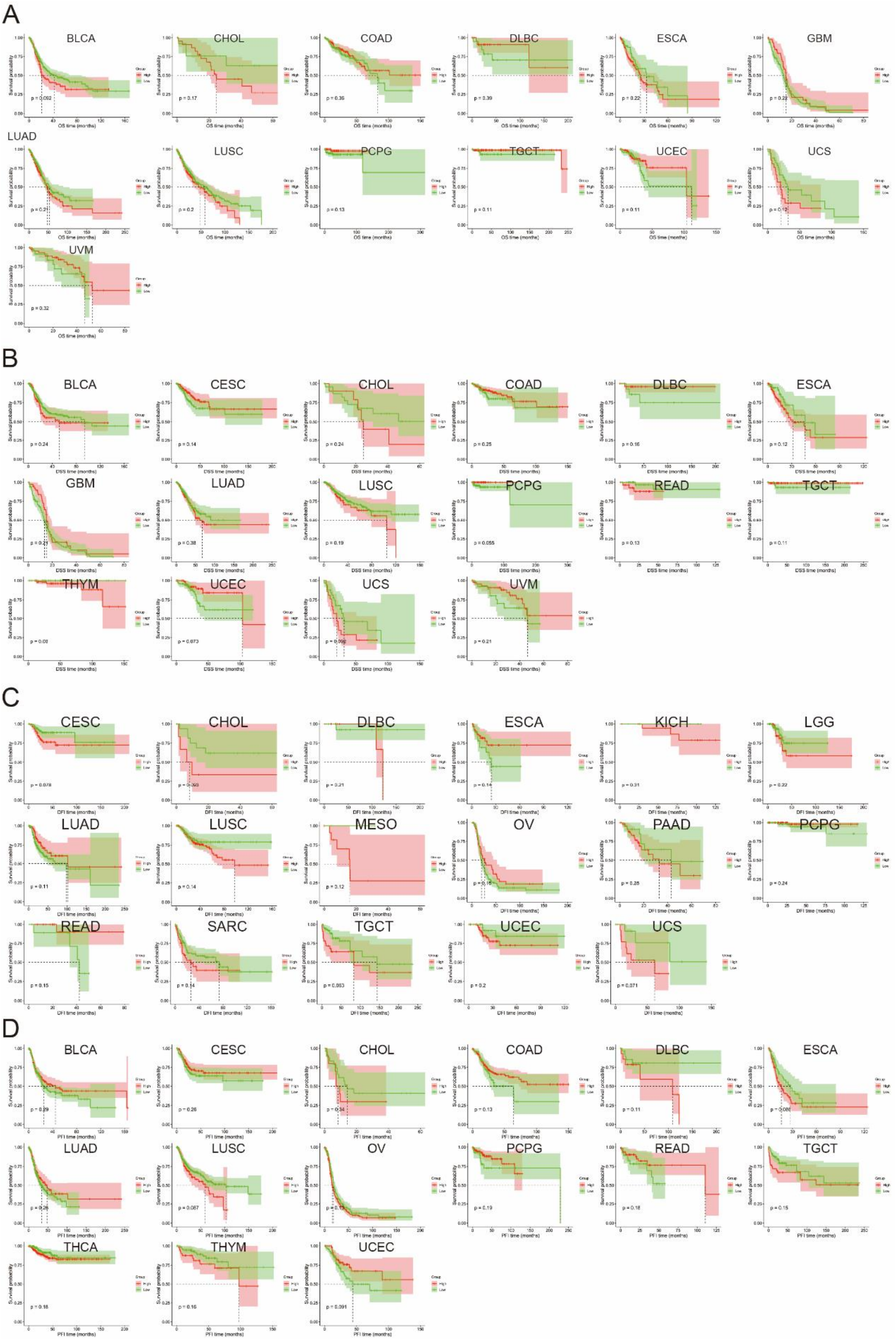

Figure S3. Kaplan-Meier survival analysis of other cancer types.

(A) OS analysis of other cancer types. **(B)** DSS analysis of other cancer types. **(C)** DFI analysis of other cancer types. **(D)** PFI analysis of other cancer types.

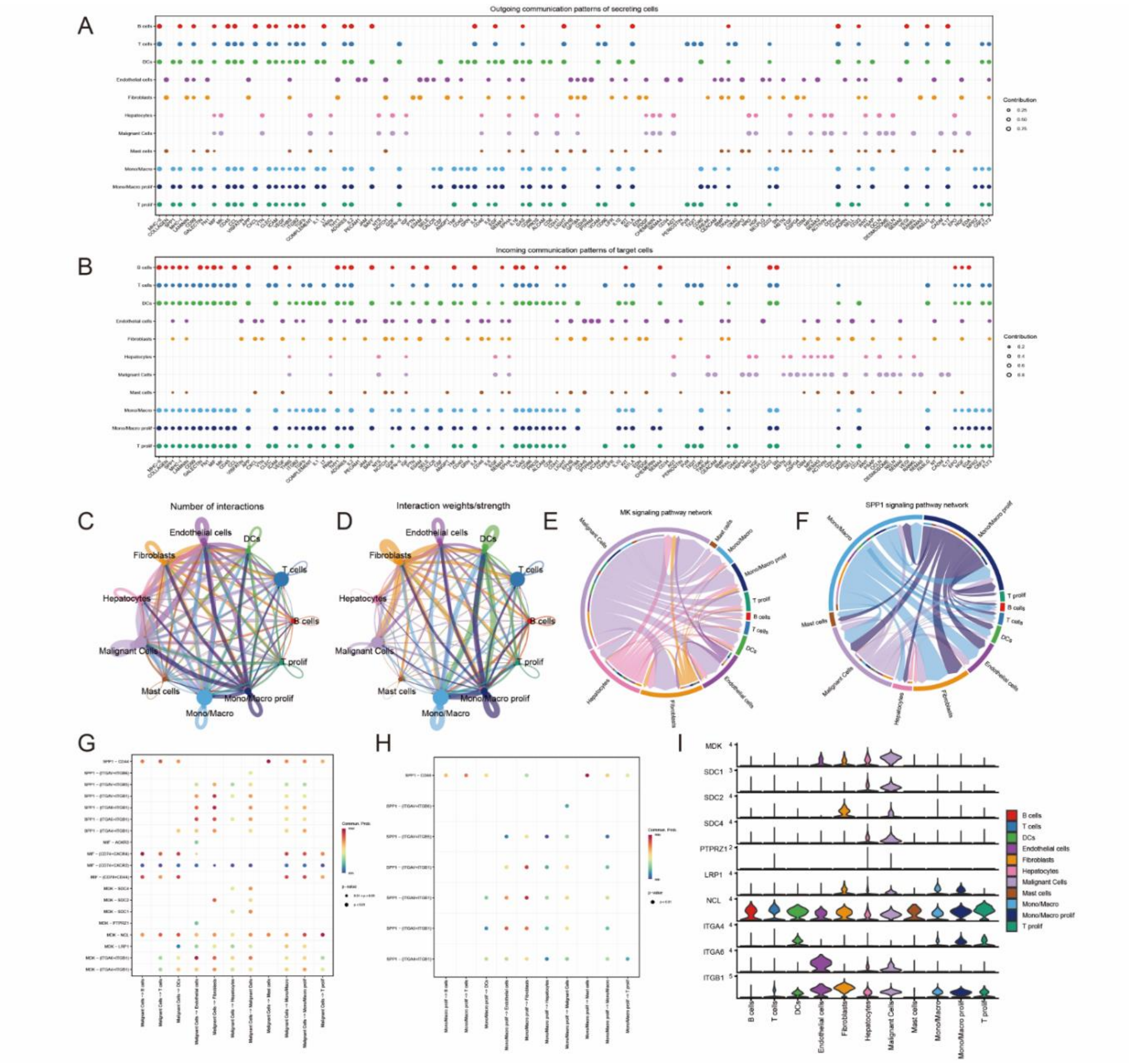

Figure S4. Cell–cell communication within the tumor microenvironment.

(A,B) Dot plots comparing the outgoing signaling patterns of secretory cells and the incoming signaling patterns of target cells. (C) Circle plot illustrating the number of communications between interacting cell types. (D) Circle plot depicting the strength of communication between interacting cell types. (E) Chord diagram showing the major cell–cell communication patterns within the MK signaling pathway. (F) Chord diagram inferring the intercellular communication network of the SPP1 signaling pathway. (G) Key ligand–receptor (LR) pairs involved in interactions between malignant cells and other cell types. (H) Key ligand–receptor pairs mediating interactions between proliferative monocytes/macrophages and other cell types. (I) Violin plot showing the expression levels of 10 genes involved in the MK signaling pathway.

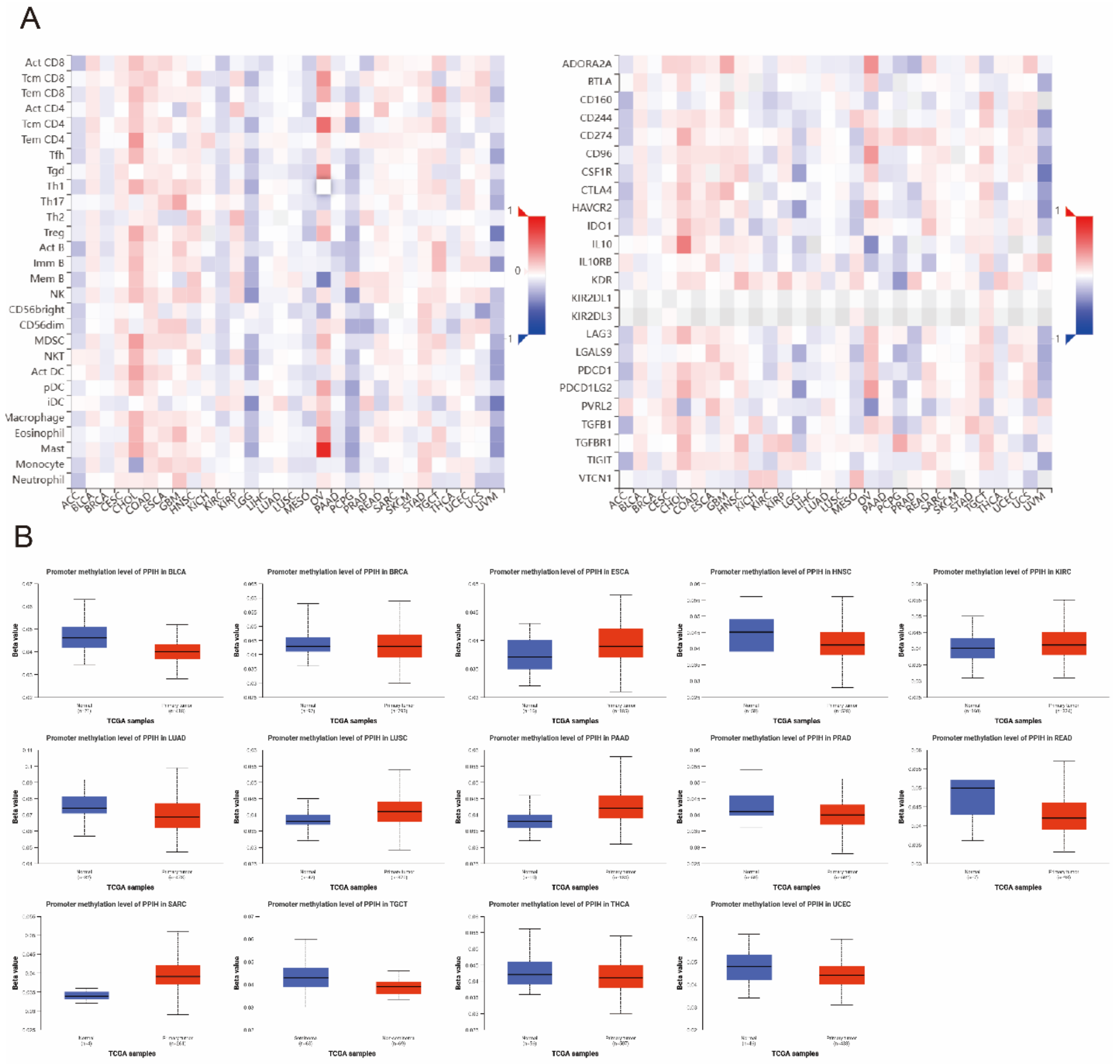

Figure S5. Methylation Analysis of PPIH

(A) Correlations of PPIH methylation degrees with immune cell infiltraions and immune checkpoint gene expressions. (B) The methylation differences of the PPIH promoter between tumor and normal tissues.

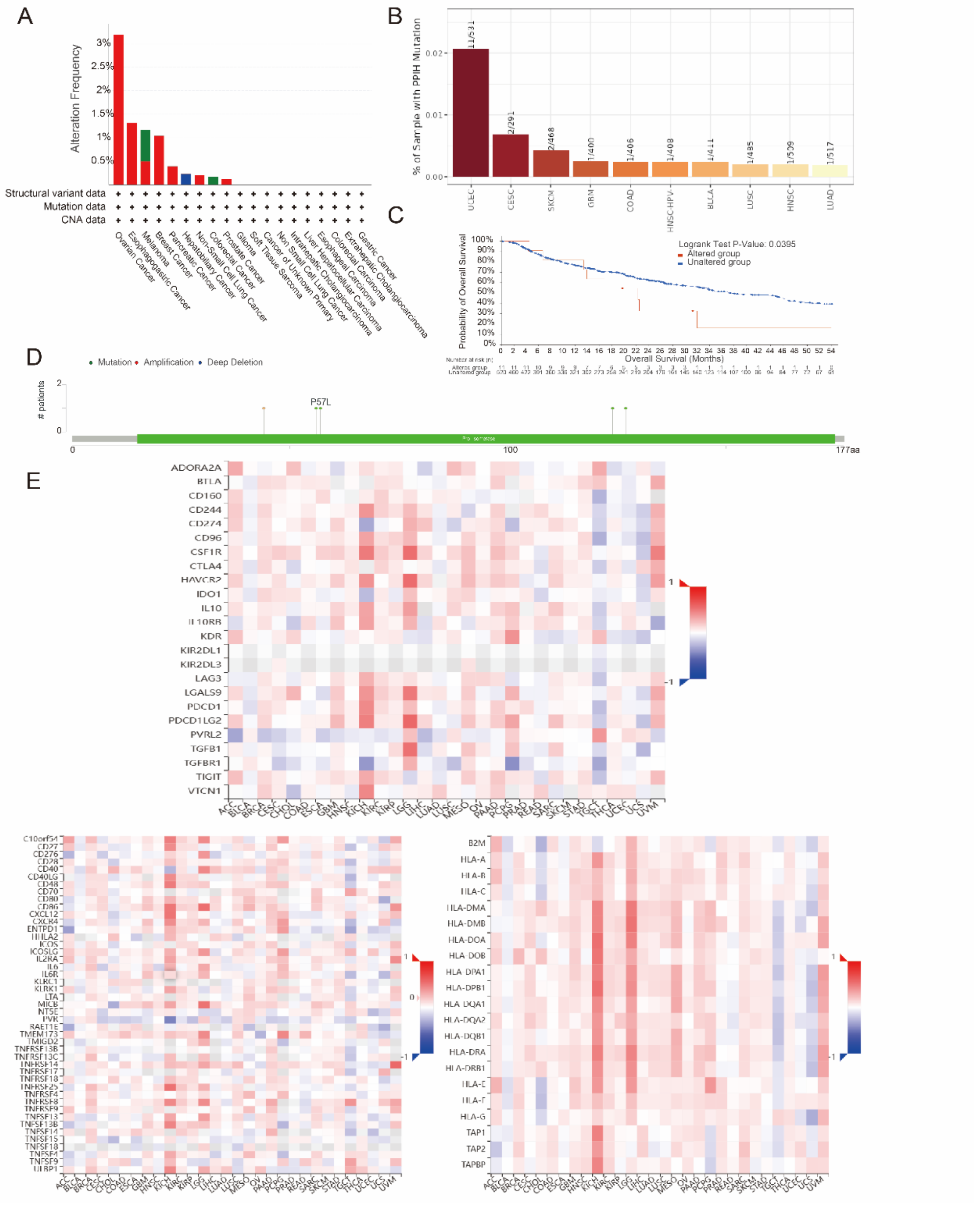

Figure S6. Genomic alteration analysis of PPIH.

(A) Genetic alteration patterns of PPIH in different types of cancers. (B) Analysis of gene mutation frequencies of PPIH in different types of cancers. (C) Kaplan-Meier curves showing the differences in OS between patients with or without PPIH mutations at the pan-cancer level. (D) Structral plot showing the different mutation sites on PPIH protien. (E) Correlations of PPIH CNV levels with the expressions of immune checkpoint genes, MHC molecules, and immunosuppressive cytokines.
